# Targeting SNRNP200-induced splicing dysregulation offers an immunotherapy opportunity for glycolytic triple-negative breast cancer

**DOI:** 10.1038/s41421-024-00715-7

**Published:** 2024-09-17

**Authors:** Wenxiao Yang, Luo Hong, Linwei Guo, Yunjin Wang, Xiangchen Han, Boyue Han, Zheng Xing, Guoliang Zhang, Hongxia Zhou, Chao Chen, Hong Ling, Zhimin Shao, Xin Hu

**Affiliations:** 1 Precision Cancer Medicine Center, Fudan University Shanghai Cancer Center, Shanghai, China; 2 Department of Breast Surgery, Fudan University Shanghai Cancer Center, Shanghai, China; 3 Key Laboratory of Breast Cancer in Shanghai, Department of Breast Surgery, Fudan University Shanghai Cancer Center, Shanghai, China; 4 Department of Oncology, Shanghai Medical College, Fudan University, Shanghai, China; 5 Department of Colorectal Surgery, Fudan University Shanghai Cancer Center, Shanghai, China; 6 Key Laboratory of Computational Biology, Shanghai Institute of Nutrition and Health, Shanghai Institutes for Biological Sciences, Chinese Academy of Sciences, Shanghai, China

**Keywords:** Breast cancer, Cancer metabolism, Cancer microenvironment, Tumour immunology

## Abstract

Metabolic dysregulation is prominent in triple-negative breast cancer (TNBC), yet therapeutic strategies targeting cancer metabolism are limited. Here, utilizing multiomics data from our TNBC cohort (*n* = 465), we demonstrated widespread splicing deregulation and increased spliceosome abundance in the glycolytic TNBC subtype. We identified SNRNP200 as a crucial mediator of glucose-driven metabolic reprogramming. Mechanistically, glucose induces acetylation at SNRNP200 K1610, preventing its proteasomal degradation. Augmented SNRNP200 then facilitates splicing key metabolic enzyme-encoding genes (*GAPDH*, *ALDOA*, and *GSS*), leading to increased lactic acid and glutathione production. Targeting SNRNP200 with antisense oligonucleotide therapy impedes tumor metabolism and enhances the efficacy of anti-PD-1 therapy by activating intratumoral CD8^+^ T cells while suppressing regulatory T cells. Clinically, higher SNRNP200 levels indicate an inferior response to immunotherapy in glycolytic TNBCs. Overall, our study revealed the intricate interplay between RNA splicing and metabolic dysregulation, suggesting an innovative combination strategy for immunotherapy in glycolytic TNBCs.

## Introduction

Triple-negative breast cancer (TNBC) is the most aggressive subtype of breast cancer and is characterized by exceedingly high rates of recurrence and mortality^1,2^. Recent advances in omics technologies have yielded crucial insights into the heterogeneity of TNBC, revealing intricate and dynamic interplay within cancer cell characteristics^3–8^. Our previous efforts focused primarily on delineating metabolic heterogeneity within TNBC subtypes, resulting in the discovery of three distinct metabolic pathway-based subtypes (MPSs): the lipogenic subtype (MPS1, characterized by heightened lipid metabolism), the glycolytic subtype (MPS2, distinguished by elevated carbohydrate and nucleotide metabolism), and the mixed subtype (MPS3, featuring partial pathway dysregulation)^4^. Furthermore, we developed individualized strategies to target intrinsic metabolic profiles tailored to specific tumor types. Despite notable advancements in the tumor response to metabolism-targeting drugs, such as lactate dehydrogenase (LDH) inhibitors in glycolytic TNBCs, their overall efficacy has been hindered by unintended impacts on noncancerous stromal and immune cells, which play critical roles in tumor progression and maintenance. Therefore, our study investigated the fundamental biological mechanisms of metabolic dysregulation in TNBC to identify more efficacious and targeted therapeutic options.

Metabolic reprogramming is recognized as a hallmark of cancer^9^. Recent studies have demonstrated that cancer cells continually reconfigure their metabolism to optimize their adaptability in response to dynamic environmental cues^10^. These adaptations rely primarily on coordinated intrinsic shifts in gene expression, orchestrating the activation or suppression of specific metabolic pathways by modulating enzyme activities^11^. RNA splicing is a key mechanism that governs gene expression, with increasing evidence supporting its crucial role in regulating cellular metabolism^12,13^. One example is the alternative splicing (AS) process that generates the pyruvate kinase M2 (PKM2) isoform of pyruvate kinase, which actively promotes glycolytic carbon flux and potentially influences cancer progression^13^. The spliceosome, a complex comprising more than 300 components, including small nuclear RNA (snRNA) molecules and proteins forming U1, U2, U4, U5, and U6 small nuclear ribonucleoprotein (snRNP) particles, orchestrates this highly regulated process^14^. Alterations in splicing-related proteins, particularly core spliceosome components, are considered a primary cause of splicing dysregulation in cancer^15,16^. Certain tumors, including TNBC, exhibit widespread splicing deregulation and heightened dependence on spliceosomal integrity, rendering them vulnerable to pharmacological splicing inhibition^17^. Modulating specific splicing genes selectively induces cell death in TNBC without affecting normal breast epithelial cells, indicating that RNA splicing is a promising therapeutic strategy^18–20^. While previous research has focused on exploring novel AS isoforms associated with disrupted cellular metabolism, a comprehensive understanding of the underlying mechanisms responsible for most splicing abnormalities in TNBC, as well as the regulation of RNA splicing in cell metabolism, remains incomplete^13,21,22^.

**Fig. 1:**
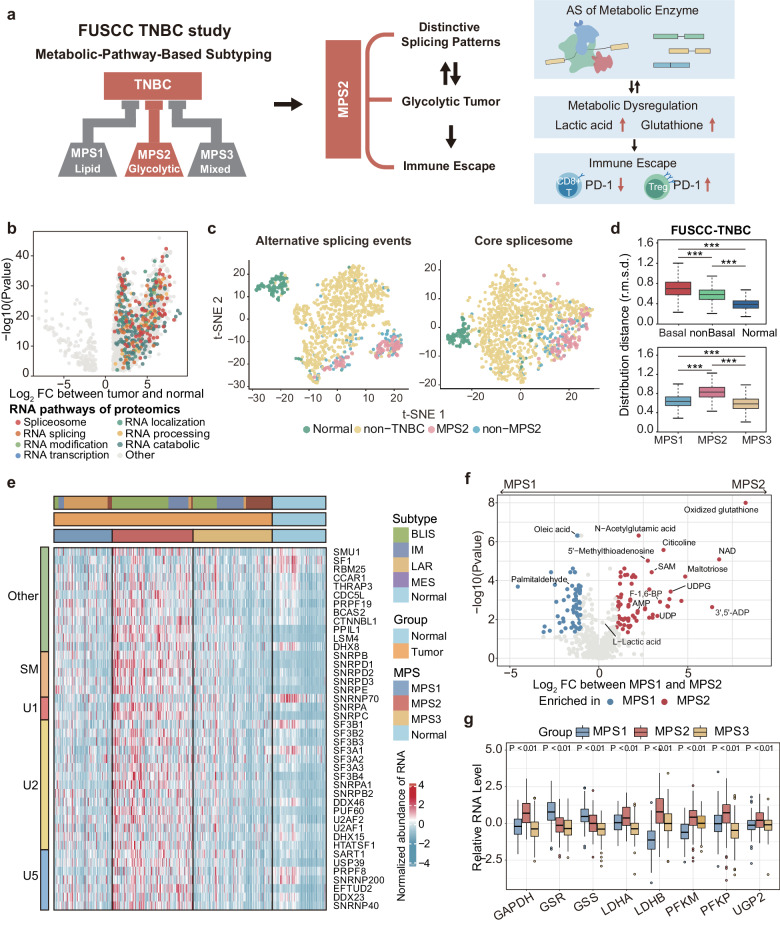
A specific landscape of alternative RNA splicing linked to TNBC metabolic dysregulation. **a** Workflow of the analytical process performed in this study. **b** Volcano plots illustrating the protein profiles of 2147 proteins displaying abnormal protein levels in TNBC (|log_2_-fold changes|>1, Benjamini‒Hochberg adjusted *P* < 0.01). A comprehensive set of 372 RNA-related proteins spanning various functional categories was meticulously color-coded for individual representation. **c** Landscape of AS for TCGA samples (*n* = 1096), as determined by splicing event PSI scores (on the left) and the expression levels of 109 core spliceosome genes (on the right). Each point corresponds to an individual sample, with color coding denoting their sample types, including normal tissues, non-TNBC, and MPSs. The position of each sample is computed as a *t*-SNE representation of the higher-dimensional splice event PSI matrix and expression matrix. **d** Top: global differences in spliceosome gene expression between basal, non-basal, and normal tissues in the FUSCC-TNBC cohort (*n* = 360). The distribution distances (r.m.s.d.) were calculated between basal tumors and corresponding normal tissues (red), non-basal tumors and corresponding normal tissues (green), and different samples of normal tissues (navy). Bottom: global differences in spliceosome gene expression between MPS subtypes in the FUSCC-TNBC cohort. The distribution distances (r.m.s.d.) were calculated between MPS1 (blue), MPS2 (red), and MPS3 (yellow) tumors and their corresponding normal tissues. *P* values were obtained from the two-sided Wilcoxon rank-sum test and the two-sided Kruskal–Wallis test (***P < 0.001). The centerline indicates the median, and the bounds of the box indicate the 25th and 75th percentiles. **e** Heatmap displaying normalized expression levels of 42 core spliceosome genes upregulated in tumors for each individual sample in the FUSCC-TNBC cohort. The sample types, four TNBC transcriptomic subtypes, and three TNBC metabolic subtypes were annotated. **f** Volcano plot of the 594 annotated metabolites between MPS1 and MPS2. Significantly differentially abundant metabolites are colored blue for upregulated MPS1 and red for upregulated MPS2. **g** mRNA expression of *GAPDH*, *GSR*, *GSS*, *LDHA*, *LDHB*, *PFKM*, *PFKP*, and *UGP2* in the three MPS subtypes in the FUSCC-TNBC cohort (Wilcoxon test). The centerline indicates the median, and the bounds of the box indicate the 25th and 75th percentiles. IM immunomodulatory subtype, LAR luminal androgen receptor, BLIS basal-like immune-suppressed subtype, MES mesenchymal-like subtype, AMP adenosine monophosphate, F-1,6-BP fructose 1,6-diphosphate, NAD nicotinamide adenine dinucleotide, SAM S-adenosylmethionine, 3’,5’-ADP adenosine 3’,5’-diphosphate.

In this study, we utilized multiomics data from our TNBC cohort to elucidate the splicing landscape of TNBC. Our findings confirmed a causal link between splicing abnormalities and metabolic dysregulation in TNBC. We pinpointed SNRNP200 as a pivotal driver in glycolytic TNBCs that promotes abnormal cellular metabolism, particularly in the lactic acid and glutathione pathways, and confers resistance to immune checkpoint blockade. Notably, we proposed and experimentally validated a combination treatment of SNRNP200 antisense oligonucleotide (ASO) and immunotherapy for glycolytic TNBCs (Fig. 1a).

## Results

### A specific landscape of alternative RNA splicing linked to TNBC metabolic dysregulation

We utilized a multiomics TNBC dataset (*n* = 465), including 360 samples with transcriptomic data, 90 paired samples with proteomic data, and 72 samples with metabolomic data, to investigate RNA splicing patterns and their role in TNBC metabolic dysregulation. We integrated gene expression and proteomic analyses to examine spliceosome shifts in TNBC and corresponding normal tissues from the Fudan University Shanghai Cancer Center (FUSCC) cohort. Proteomic analysis identified 372 abnormally upregulated RNA-related proteins in TNBC tissues, including 141 involved in RNA splicing and 42 core spliceosome components (Fig. 1b and Supplementary Table S1). Principal component analysis (PCA) revealed distinct core spliceosome gene expression profiles in TNBC samples compared with non-TNBC and normal samples in both the FUSCC and TCGA cohorts (Supplementary Fig. S1a). *t*-distributed stochastic neighbor embedding (*t*-SNE) further underscored the diversity in RNA splicing characteristics among TNBC subtypes (Fig. 1c). Euclidean distance analysis indicated that TNBCs, particularly the MPS2 subtype, exhibit substantial transcriptomic diversity in spliceosome gene expression (Fig. 1d and Supplementary Fig. S1b–e). The glycolytic MPS2 subtype, which is associated with higher tumor grades and poorer prognosis, exhibited unique splicing patterns and elevated expression of 42 core spliceosome components, particularly the U1, U2, and U5 snRNP genes (Fig. 1e). Validation with an additional cohort of 179 transcriptomic samples from the Precision Cancer Medicine Center (PCMC) and TCGA cohorts confirmed that the glycolytic MPS2 subtype exhibited a distinct alternative RNA splicing landscape consistent with FUSCC-TNBC findings (Supplementary Fig. S1f).

Metabolomic data revealed significant accumulation of glycolysis and nucleotide metabolism intermediates, including lactic acid, oxidized glutathione, fructose 1,6-diphosphate (F-1,6-BP), adenosine diphosphate (ADP), uridine diphosphate glucose (UDPG), and S-adenosylmethionine (SAM), in MPS2 tumors (Fig. 1f and Supplementary Table S2). This accumulation corresponded with notable increases in the RNA levels of key metabolic enzymes responsible for synthesizing and decomposing these metabolites, indicating the crucial role of RNA in regulating TNBC metabolism (Fig. 1g and Supplementary Fig. S1g). Overall, TNBC subtypes displayed distinct RNA splicing patterns, with glycolytic tumors showing upregulated core spliceosome genes and unique RNA splicing profiles, suggesting vulnerability to splicing inhibition. However, the mechanism by which glycolytic tumors activate RNA splicing to reprogram metabolism remains unknown and warrants further investigation into RNA splicing regulation for therapeutic potential.

### Identification of SNRNP200 as a crucial regulator that fuels glycolytic TNBCs in vitro and in vivo

**Fig. 2:**
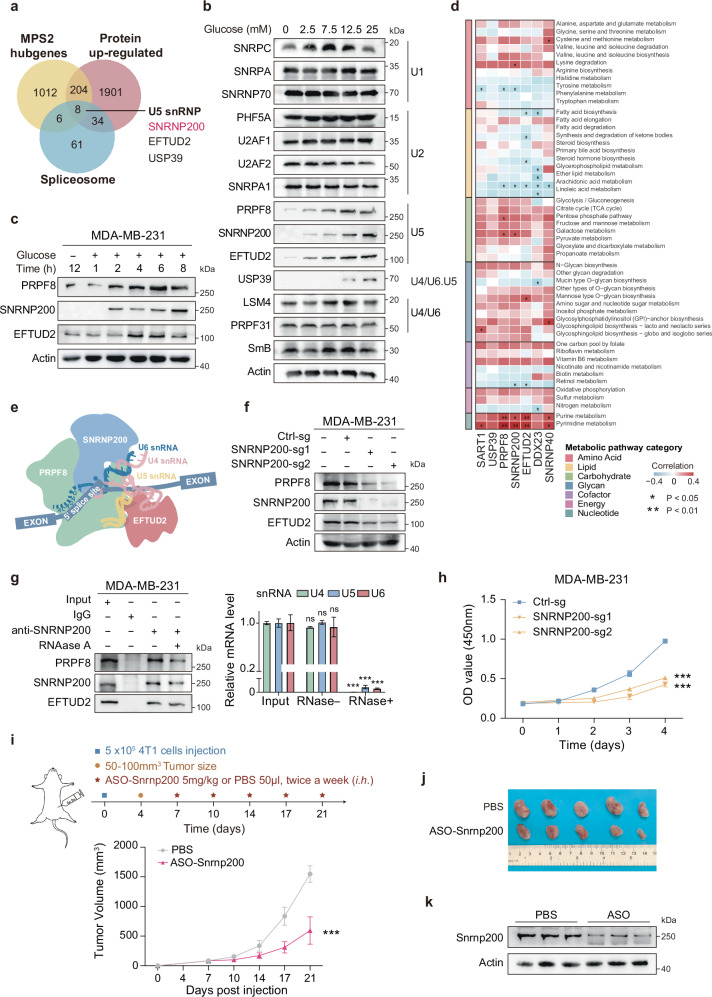
SNRNP200 is a crucial regulator of glycolytic TNBCs, promoting tumor proliferation both in vitro and in vivo. **a** Venn diagram depicting the overlap between the MPS2 hub genes and the 42 spliceosome genes whose protein levels were upregulated in TNBC. **b** MDA-MB-231 cells were cultured in media supplemented with glucose at the indicated concentrations for 16 h. The cell lysates were subjected to immunoblotting. **c** MDA-MB-231 cells were glucose-starved for 12 h and then stimulated with glucose (25 mM) for the indicated times. The cell lysates were subjected to immunoblotting. **d** Correlation coefficient heatmap illustrating associations between normalized mRNA expression levels of upregulated U5 snRNP genes in tumors and enrichment scores of metabolic pathways via Spearman’s correlation analysis in the FUSCC-TNBC cohort (*P < 0.05; **P < 0.01). **e** Diagram depicting the U5 snRNP core components. **f** Western blot analysis of SNRNP200, EFTUD2, and PRPF8 expression levels in control and SNRNP200-knockdown MDA-MB-231 cells. **g** MDA-MB-231 cells were immunoprecipitated with an anti-SNRNP200 antibody, with IgG serving as a negative control. The IP data were analyzed by western blot analysis (on the left). qPCR analysis of the U4, U5, and U6 snRNA levels in the input, RNase A^−^, and RNase A^+^ groups. The relative U4, U5, and U6 snRNA levels were normalized to that of the input using the 2^−Ct^ method (on the right). qPCR analysis data are presented as the mean ± SEM. **h** CCK-8 proliferation assay in control and SNRNP200-knockdown MDA-MB-231 cells. **i** Schematic outline showing the *Snrnp200*-targeted ASO treatment timeline: 4T1 mouse breast cancer cells were subcutaneously injected into BALB/c mice. When the tumors reached 50–100 mm^3^, the mice were treated with ASO-*Snrnp200* (5 mg/kg subcutaneous injection, twice a week) or PBS (50 µL, subcutaneous injection, twice a week) for 2 weeks (*n* = 5 mice/group). Tumor growth in different groups. The data are presented as the mean ± SEM. **j** A representative image of 4T1 tumors illustrating the effect of ASO-*Snrnp200* treatment (*n* = 5 mice/group). **k** Western blot analysis of mouse Snrnp200 protein expression in tumor tissues from 4T1 cell-derived xenografts. The data are representative of three independent biological replicates. For **g**–**i**, the data were compared using Student’s *t*-test if the data in each group were normally distributed: ***P < 0.001; ns not significant, *P* > 0.05.

Using weighted gene coexpression network analysis (WGCNA), we identified 1230 genes showing significant and coordinated alterations in the MPS2 subtype (Supplementary Fig. S2a). Among these genes, 8 overlapped with 42 upregulated core spliceosome proteins, including 2 U5 snRNP components (SNRNP200 and EFTUD2) and 1 U4/U6.U5 tri-snRNP component (USP39), indicating a regulatory role for U5 snRNPs in the glycolytic subtype (Fig. 2a). To explore metabolic regulation by spliceosome components in MPS2, we investigated their response to glucose variations. A screen of 14 core spliceosome proteins revealed that U5 snRNP component levels were significantly sensitive to glucose fluctuations in MPS2 cells (MDA-MB-231 and BT-549), whereas mRNA levels remained stable (Fig. 2b and Supplementary Fig. S2b, c). Notably, USP39 levels varied at both the RNA and protein levels under different glucose conditions, suggesting distinct regulatory mechanisms (Supplementary Fig. S2d). Glucose deprivation experiments confirmed the impact of glucose availability on U5 snRNPs, with the reintroduction of glucose significantly increasing protein levels (Fig. 2c and Supplementary Fig. S2e). Moreover, under high-glucose conditions (25 mM), the half-life of U5 snRNP proteins was extended (Supplementary Fig. S2f). In MPS2 breast cancer cells, there was a consistent and significant increase in the expression of these crucial proteins, with SNRNP200 exhibiting the most pronounced fluctuations in response to glucose concentrations (Fig. 2b, c and Supplementary Fig. S2b–f). By contrast, the U5 snRNP protein levels of the TNBC subtypes MPS1 (MDA-MB-468) and MPS3 (HCC1143) exhibited minor changes in response to glucose fluctuations compared with those of MPS2 (Supplementary Fig. S2g–i), indicating subtype-specific metabolic dependencies. The core U5 snRNP components were consistently upregulated in MPS2 TNBC cells and across TNBC patient cohorts (Supplementary Fig. S3a, b). Multiplex immunohistochemistry (mIHC) also confirmed the upregulation of U5 snRNP proteins in MPS2 TNBCs (Supplementary Fig. S4a). Gene set variation analysis (GSVA) further revealed positive correlations between carbohydrate and nucleic acid metabolism pathways (dominant features of MPS2) and the RNA abundance of U5 snRNP components, whereas lipid metabolism was negatively correlated (Fig. 2d and Supplementary Fig. S3c). These findings suggest that glucose-stabilized U5 snRNP proteins, coupled with increased RNA levels, contribute to the dysregulated metabolism observed in the MPS2 subtype of TNBC.

The U5 snRNP is crucial for spliceosome activation and active site formation because its catalytic function is linked to the PRPF8, SNRNP200, and EFTUD2 proteins^23^. In SNRNP200-knockdown cells, the PRPF8 and EFTUD2 protein levels decreased without affecting the corresponding mRNA levels, indicating that SNRNP200 stabilizes the U5 complex independently of transcription (Fig. 2f and Supplementary Fig. S5a–c). Immunoprecipitation (IP) assays confirmed that stable protein‒protein interactions between SNRNP200, PRPF8, and EFTUD2 were unaffected by U5 snRNA digestion (Fig. 2g and Supplementary Fig. S5d). Cycloheximide (CHX) treatment further confirmed that SNRNP200 knockdown led to shortened half-lives of PRPF8 and EFTUD2. Introduction of an SNRNP200 mutant (SNRNP200^Mut^) resistant to targeting by *SNRNP200* sgRNA-1 restored protein levels and extended half-lives under high glucose conditions, confirming the role of SNRNP200 in U5 complex stability (Supplementary Fig. S5e–g). Furthermore, compared with normal breast epithelial cells (MCF10A), TNBC cells, particularly those with glycolytic subtypes (Hs-578T, BT-549, and MDA-MB-231), presented significantly elevated SNRNP200 mRNA and protein levels (Supplementary Fig. S5h, i). In clinical samples, SNRNP200 was markedly upregulated in glycolytic TNBC subtypes and correlated with poorer overall survival (Supplementary Fig. S5j, k).

To investigate the biological function of SNRNP200, we conducted SNRNP200 knockdown experiments in MPS2 cells via CRISPR-Cas9 technology. Colony formation, EdU incorporation, and CCK-8 assays revealed significant inhibition of MPS2 cell proliferation in vitro following SNRNP200 knockdown (Fig. 2h and Supplementary Fig. S6a–e). Flow cytometry analysis revealed increased early- and late-stage apoptosis in SNRNP200-knockdown MPS2 cells (Supplementary Fig. S6f, g). Conversely, SNRNP200 knockdown in MCF10A cells resulted in minimal growth reduction, highlighting the selective vulnerability of glycolytic cancer cells to SNRNP200 depletion (Supplementary Fig. S6h–j). To explore SNRNP200 as a potential therapeutic target for glycolytic TNBC, we tested a panel of ASO agents targeting mouse *Snrnp200*. Among the three designed ASOs, ASO-2 achieved the greatest reduction in Snrnp200 expression, whereas control ASOs had no effect on Snrnp200 levels in a murine MPS2 cell line (4T1; Supplementary Fig. S6k, l). Treatment of 4T1 cells with ASO-*Snrnp200* significantly inhibited cell growth and increased apoptosis in vitro (Supplementary Fig. S6m–p). To evaluate the in vivo efficacy of ASO-*Snrnp200*, we generated 4T1 xenograft tumors by injecting 5 × 10^5^ 4T mouse breast cancer cell lines into mice treated with ASO-*Snrnp200*, which exhibited a substantial reduction in tumor growth compared with that in PBS-treated controls (Fig. 2i, j). On-target pharmacodynamic activity was confirmed by reduced Snrnp200 protein expression in tumors (Fig. 2k).

### Elevated glucose levels trigger SNRNP200 K1610 acetylation, safeguarding it from proteasomal degradation

**Fig. 3:**
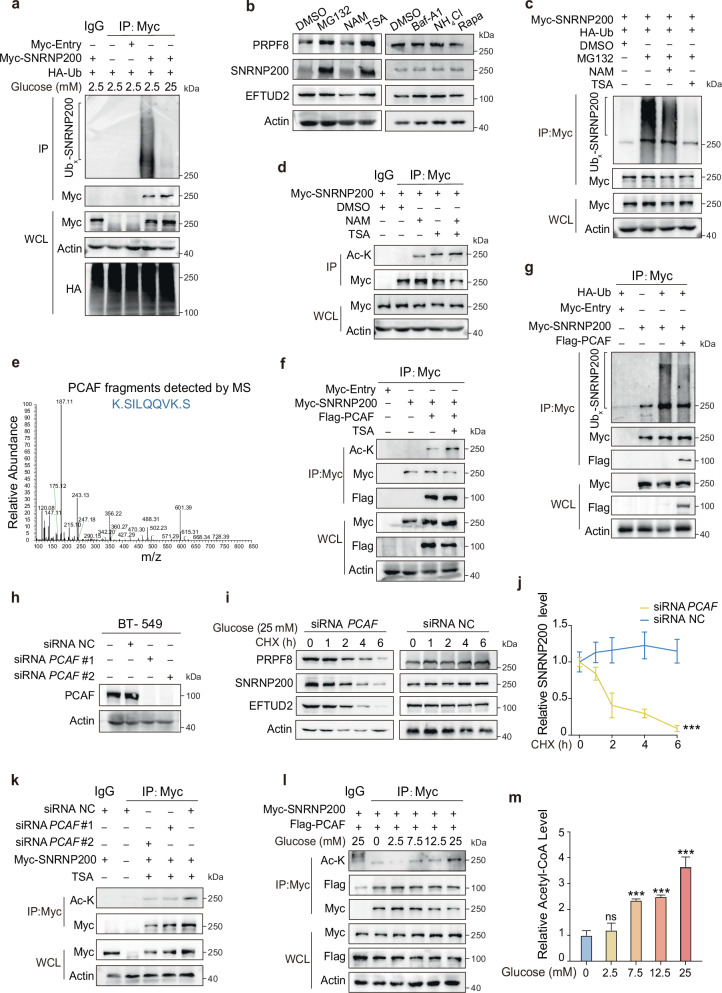
Elevated glucose levels trigger PCAF-mediated acetylation of SNRNP200. **a** HEK293T cells were transfected with Myc-tagged SNRNP200 and HA-Ub as indicated and treated with glucose at either 2.5 mM or 25 mM. The cells were harvested for ubiquitylation analysis. **b** Immunoblot analysis of SNRNP200, EFTUD2, and PRPF8 protein levels in BT-549 cells treated with or without nicotinamide (NAM, 5 mM, 6 h), TSA (10 μM, 16 h), Baf-A1 (200 nM, 16 h), NH_4_Cl (20 mM, 16 h), or Rapa (1 μM, 16 h). MG132 treatment (10 μM, 12 h) was used as a positive control. **c,** **d** After treatment with or without NAM, TSA, or MG132, the ubiquitylation levels (**c**) and acetylation levels (**d**) of SNRNP200 in HEK293T cells were measured by immunoblotting with the indicated antibodies. **e** Endogenous PCAF was immunoprecipitated from HEK293T cells with an anti-SNRNP200 antibody and analyzed by LC-MS. The data show MS2 spectra for a signature peptide of PCAF. **f** Myc-SNRNP200 was cotransfected with Flag-tagged PCAF into HEK293T cells. Acetylation was determined by immunoblotting. Co-IP assays were performed to determine the interaction between SNRNP200 and PCAF. **g** Flag-tagged PCAF was cotransfected with Myc-tagged SNRNP200 and HA-tagged ubiquitin. The ubiquitylation of SNRNP200 was determined by IP-western blotting with an anti-HA antibody. **h** Western blot analysis of PCAF expression levels in control and PCAF-depleted BT-549 cells. **i**, **j** BT-549 cells maintained in 25 mM glucose were transfected with si*PCAF* or the control and treated with CHX as previously described. The endogenous SNRNP200, EFTUD2, and PRPF8 proteins were analyzed by immunoblotting (**i**) and quantified against actin (**j**). The data for the relative SNRNP200 levels are presented as the mean ± SEM. **k** BT-549 cells were transfected with si*PCAF* or the control. The acetylation levels of SNRNP200 were analyzed by immunoblotting. **l** BT-549 cells were treated with glucose at the indicated concentrations. The acetylation levels of SNRNP200 were analyzed by immunoblotting. Co-IP assays were performed to determine the dynamic interactions between PCAF and SNRNP200. **m** Acetyl-CoA levels were measured in BT-549 cells treated with glucose at the indicated concentrations. The relative acetyl-CoA levels are presented as the mean ± SEM. For **j** and **m**, the data were compared using Student’s *t*-test: ns not significant, *P* > 0.05; ***P < 0.001.

Under high-glucose conditions, we observed an increase in the SNRNP200 protein level and the level of stable RNA (Fig. 2b, c and Supplementary Fig. S2b, c). Given the roles of the ubiquitin-proteasome system and autophagy in cellular degradation, we investigated the degradation pathway of SNRNP200. In vivo, ubiquitylation assays in MPS2 cells revealed a marked decrease in SNRNP200 ubiquitylation under high-glucose conditions (Fig. 3a). Treatment with the proteasome inhibitor MG132 or the histone deacetylase inhibitor trichostatin A (TSA) increased SNRNP200 protein levels (Fig. 3b and Supplementary Fig. S7a). However, autophagy inhibitors such as bafilomycin A1 (Baf-A1) and ammonium chloride (NH_4_Cl), as well as the autophagy inducer rapamycin (Rapa), did not affect SNRNP200 levels in MPS2 cells. These findings collectively indicate that the degradation of SNRNP200 primarily occurs through the ubiquitin-proteasome pathway rather than the autophagy-lysosome pathway. Moreover, TSA treatment significantly augmented SNRNP200 acetylation, concurrently impeding its ubiquitylation (Fig. 3c, d). IP and liquid chromatography-mass spectrometry (IP-LC-MS) data identified PCAF as a potential acetyltransferase responsible for SNRNP200 acetylation (Fig. 3e). The interaction between SNRNP200 and PCAF was further validated through IP assays (Fig. 3f and Supplementary Fig. S7b). Moreover, PCAF overexpression enhanced SNRNP200 acetylation and inhibited its ubiquitylation, whereas endogenous PCAF depletion led to a notable decrease in SNRNP200 acetylation, a significant reduction in the half-life of SNRNP200, and a subsequent decrease in the protein level of SNRNP200 (Fig. 3f–k). Notably, in vitro experiments indicated that glucose did not directly influence the interaction between PCAF and SNRNP200 but likely affected the accessibility of other contributing factors (Fig. 3l). Recent studies have shown that acetylation is directly linked to acetyl-CoA and is affected by acetyl-CoA availability^24,25^. Consistently, we observed significant fluctuations in the intracellular acetyl-CoA levels corresponding to changes in the glucose concentration, which is consistent with the observed pattern of SNRNP200 acetylation (Fig. 3l, m).

**Fig. 4:**
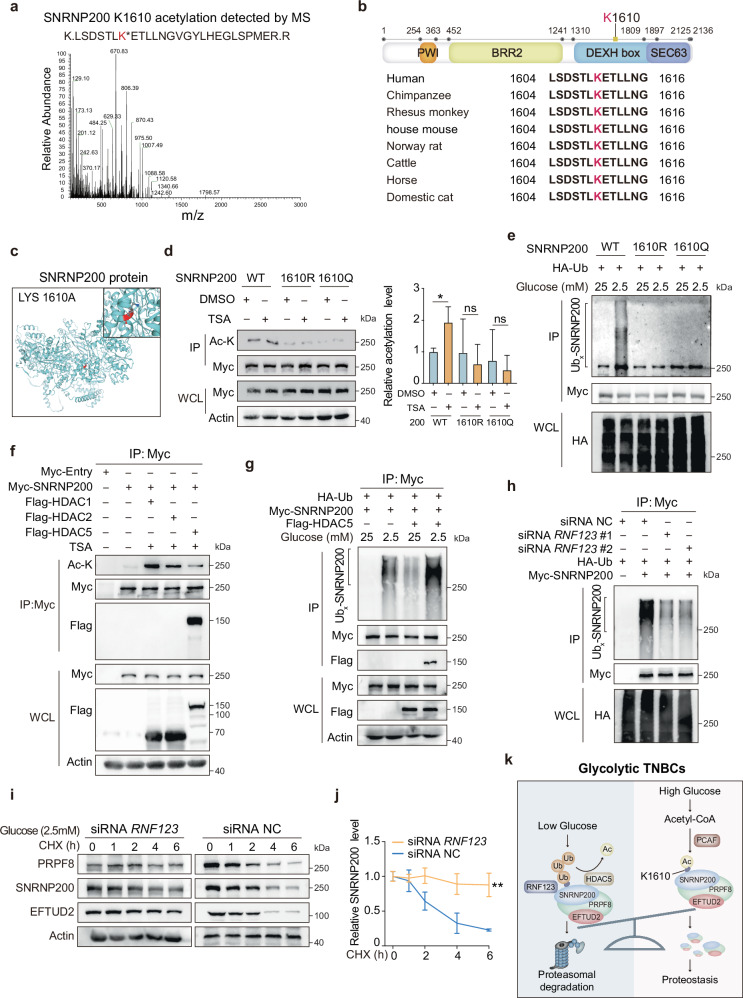
The acetylation of SNRNP200 at lysine 1610 safeguards it from proteasomal degradation. **a** Endogenous SNRNP200 was immunoprecipitated from HEK293T cells and analyzed by LC-MS. The data show MS2 spectra for a signature peptide acetylated at K1610. **b** Alignment of the SNRNP200 protein sequence across different species. **c** A secondary structure model of SNRNP200 was visualized via SWISS-MODEL^68^. The K1610 residue is highlighted with a different color. **d** HEK293T cells were transfected with Myc-tagged SNRNP200 WT, K1610R, or K1610Q plasmids for 36 h with or without TSA. The acetylation levels of SNRNP200 were quantified against immunoprecipitated Myc-tag and are presented as the mean ± SEM. **e** HEK293T cells were transfected with the indicated plasmids and treated with glucose at either 2.5 mM or 25 mM. Ubiquitylation analysis was revealed by immunoblotting. **f** HDAC1, HDAC2, and HDAC5 were overexpressed in HEK293T cells treated with or without TSA. The acetylation levels of SNRNP200 were determined by immunoblotting. Co-IP assays were performed to determine the interaction between SNRNP200 and HDAC5. **g** HEK293T cells were maintained in a medium supplemented with either 2.5 mM or 25 mM glucose. The SNRNP200 ubiquitylation levels were determined via IP-western blotting. Co-IP assays were performed to determine the dynamic interactions between SNRNP200 and HDAC5. **h** BT-549 cells were transfected with si*RNF123* or the control. The levels of ubiquitinated SNRNP200 were analyzed by immunoblotting. **i**, **j** BT-549 cells maintained in 2.5 mM glucose were transfected with si*RNF123* or the control and treated with CHX as previously described. The endogenous SNRNP200, EFTUD2, and PRPF8 proteins were analyzed by immunoblotting (**i**) and quantified against actin (**j**). The data for the relative SNRNP200 levels are presented as the mean ± SEM. **k** Working model illustrating the mutually exclusive acetylation and ubiquitylation of SNRNP200 in glycolytic TNBCs. For **d** and **j**, the data were compared using Student’s *t*-test: ns not significant, *P* > 0.05; *P < 0.05; **P < 0.01.

IP-LC-MS data identified lysine 1610 (K1610) as the primary acetylation site on SNRNP200 (Fig. 4a). K1610 is located within the DEXH box domain (residues 1310–1897) and is highly conserved across species (Fig. 4b, c). TSA treatment increased acetylation and decreased the ubiquitylation of wild-type (WT) SNRNP200 but not of the K1610R (unacetylated) or K1610Q (acetylation mimicked) mutants (Fig. 4d and Supplementary Fig. S7c, d). High glucose stabilized WT SNRNP200 but not the K1610R or K1610Q mutants (Fig. 4e). To determine the deacetylase responsible for SNRNP200, we investigated three prominent deacetylases and found that histone deacetylase 5 (HDAC5) specifically facilitated SNRNP200 deacetylation (Fig. 4f). HDAC5 overexpression decreased SNRNP200 acetylation and increased ubiquitylation (Fig. 4f, g). Endogenous IP assays confirmed the interaction between SNRNP200 and HDAC5 in MPS2 tumor cells (Supplementary Fig. S7e). Moreover, HDAC5 exhibited a greater affinity for SNRNP200 exposed to lower glucose concentrations (2.5 mM), with diminished binding occurring as the glucose concentration increased (Fig. 4g). RNF123 was identified as a potential SNRNP200-interacting protein via IP-MS (Supplementary Fig. S7f). This interaction was validated in HEK293T and MPS2 cells (Supplementary Fig. S7g, h). Lowering glucose increased SNRNP200 ubiquitylation and its association with RNF123 (Supplementary Fig. S7i). RNF123 depletion decreased SNRNP200 ubiquitylation under low-glucose conditions, extending the half-life of SNRNP200 (Fig. 4h–j and Supplementary Fig. S7j). Notably, low glucose effectively promoted K48-type polyubiquitylation, whereas monoubiquitylation or the non-degradative K63-type polyubiquitylation of SNRNP200 was not observed (Supplementary Fig. S7k). These findings indicated that glucose protected SNRNP200 from proteasomal degradation.

In summary, our data revealed that increased glucose uptake in glycolytic subtypes elevates acetyl-CoA levels, maintaining SNRNP200 K1610 acetylation through PCAF. This prevents HDAC5-mediated deacetylation and RNF123-mediated K48-type polyubiquitylation, protecting SNRNP200 from proteasomal degradation (Fig. 4k).

### SNRNP200 enhances RNA splicing in metabolic enzyme-encoding genes with weak 5’ splice sites

**Fig. 5:**
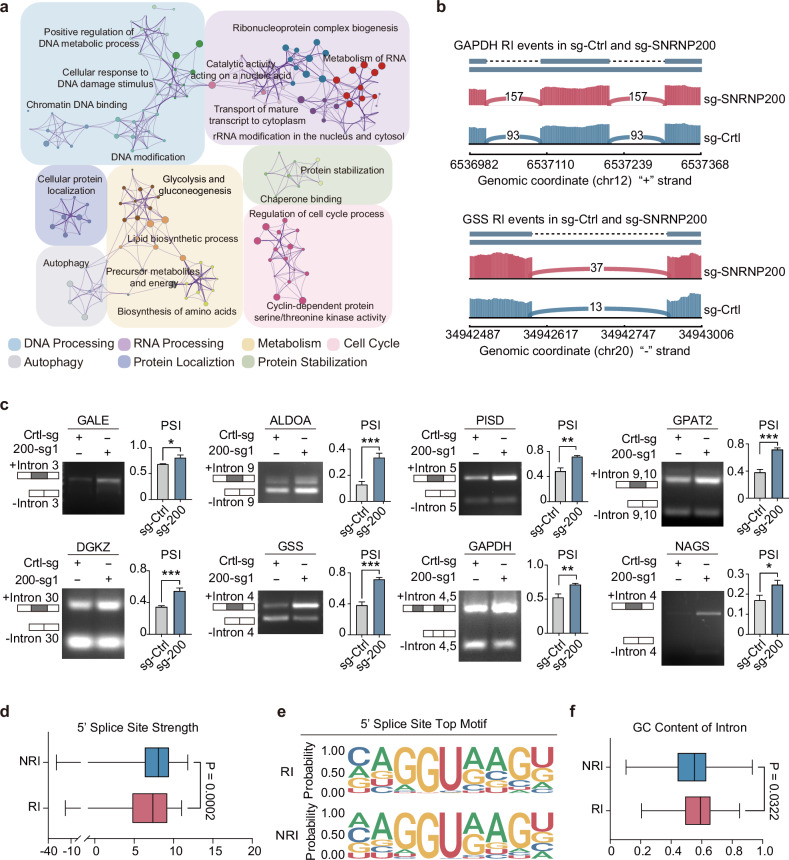
SNRNP200 enhances RNA splicing in genes encoding metabolic enzymes with weak 5’ splice sites. **a** Enrichment networks of intron-retained transcripts in control and SNRNP200-knockdown MDA-MB-231 cells were analyzed and visualized by Metascape. Representative terms with kappa similarities above 0.3 formed a network and were depicted using Cytoscape software. **b** Sashimi plots depicting the RIs of *GAPDH* (top) and *GSS* (bottom). The number of RI reads is indicated (control sgRNA in blue, *SNRNP200* sgRNA in red). **c** Representative RT-PCR validation of SNRNP200-regulated RI events in MDA-MB-231 cells. The structure of each isoform is illustrated in the diagrams. Individual data points are presented (*n* = 3; *P < 0.05; *P < 0.01; **P < 0.001; Student’s *t*-test). **d** A 5’ splice site strength analysis of RI events was carried out. Non-retained introns (NRIs) within the same set of genes were used for comparison (Wilcoxon rank-sum test). **e** Motif enrichment analysis of the same set of genes revealed that the most frequently identified motifs aligned with the consensus 5’ splice site sequences for both the RI and NRI. **f** The GC compositions of the RI and NRI were calculated by dividing the GC content of each intron by the average of its adjacent exons and compared by the Wilcoxon rank-sum test.

We employed RNA sequencing (RNA-seq) analysis to investigate SNRNP200-mediated splicing regulation within the glycolytic subtype of TNBC. SNRNP200 knockdown led to significant alterations in 2742 AS events across 2134 genes (FDR < 0.05 and ΔPSI > 0.02), including skipped exons (SEs), alternative 5’ splice sites (A5SSs), alternative 3’ splice sites (A3SSs), retained introns (RIs), and mutually exclusive exons (MXEs; Supplementary Fig. S8a). Notably, 292 genes presented two or more AS events, highlighting the impact of SNRNP200 on transcriptome diversity. A greater proportion of RI events were upregulated than other AS types, affecting introns enriched in genes linked to cell cycle regulation, DNA/RNA processing, and cellular metabolism (Fig. 5a and Supplementary Fig. S8b and Table S3). Specifically, metabolic enzymes involved in glycolysis and nucleotide metabolism displayed altered splicing patterns upon SNRNP200 depletion. For example, GAPDH, a key glycolysis enzyme responsible for converting glyceraldehyde 3-phosphate to 1,3-diphosphoglycerate, exhibited altered RNA splicing following SNRNP200 knockdown, resulting in the retention of introns 4 and 5. Similarly, *GSS* transcripts encoding glutathione (GSH) synthase were enriched in intron 4 after SNRNP200 depletion (Fig. 5b). Semiquantitative RT-PCR validated the role of SNRNP200 in modulating the splicing of representative genes (Fig. 5c and Supplementary Fig. S8c).

We further investigated features common to transcripts with increased RI events, focusing on differences in splice site strength between retained and properly spliced introns post-SNRNP200 depletion. Our analysis revealed that introns with inefficient splicing tended to have weaker 5’ splice sites (*P* value = 0.0002, Wilcoxon test; Fig. 5d), whereas distinctions at the 3’ splice site were less pronounced (*P* value = 0.2932, Wilcoxon test; Supplementary Fig. S8d). Motif enrichment analysis supported these findings (Fig. 5e and Supplementary Fig. S8e). Additionally, compared with non-RIs, RIs presented a slightly greater GC content, resembling the GC levels observed in adjacent exons (Fig. 5f). Taken together, these findings underscore the critical role of U5 snRNP components, particularly SNRNP200, in regulating the splicing patterns of metabolic enzyme-encoding genes characterized by weak 5’ splice sites.

### SNRNP200 enhances glycolysis and glutathione metabolism via RNA splicing

**Fig. 6:**
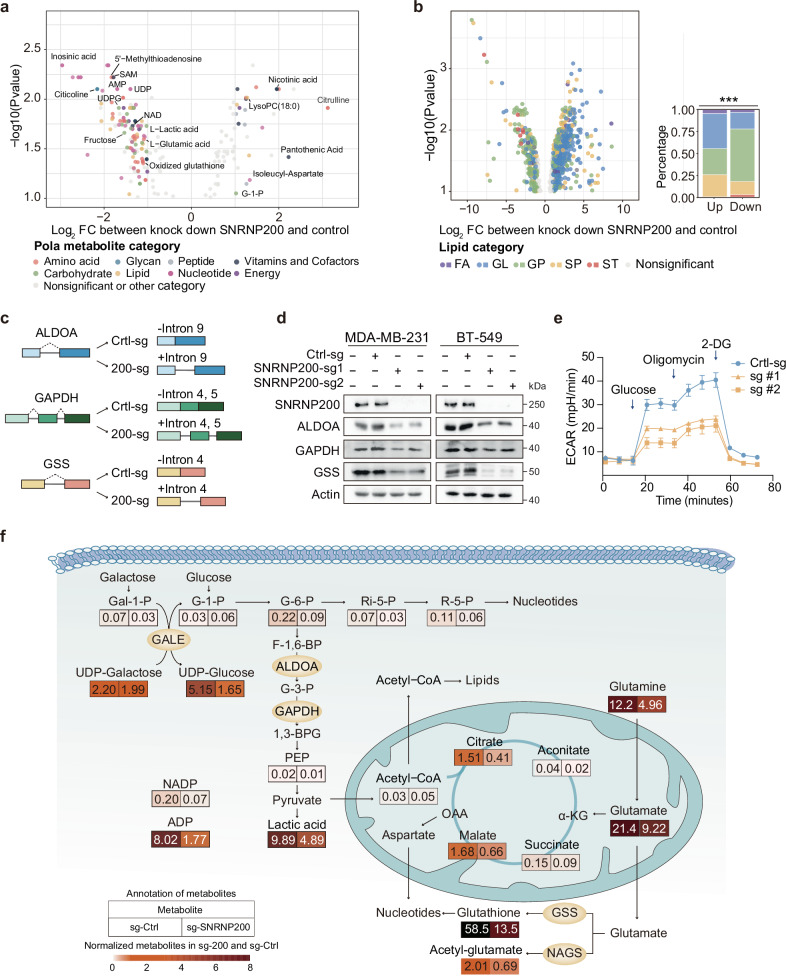
SNRNP200 enhances glycolysis and glutathione metabolism via RNA splicing. **a** Volcano plot of the polar metabolites profiled in control and SNRNP200-knockdown MDA-MB-231 cells. Significantly differentially abundant metabolites are color-coded by individual category. **b** Volcano plot of the lipid profiles of control and SNRNP200-knockdown MDA-MB-231 cells. Metabolites with significant differences are color-coded according to their respective categories. Furthermore, the proportions of five distinct lipid categories in SNRNP200-knockdown MDA-MB-231 cells were assessed. The data were statistically analyzed using the chi-square test (***P < 0.001). **c** Images of the RIs of *ALDOA*, *GAPDH*, and *GSS*. **d** Western blot analysis of the expression levels of SNRNP200, ALDOA, GAPDH, and GSS in control and SNRNP200-knockdown MDA-MB-231 and BT-549 cells. **e** Comparison of the ECAR between control and SNRNP200-knockdown MDA-MB-231 cells. **f** Diagram summarizing metabolic genes involved in glycolysis, the TCA cycle, and glutamate metabolism. The diagram visually depicts normalized metabolite levels (boxes) and their respective upstream metabolic enzymes (circles) undergoing intron retention induced by SNRNP200 ablation. UDP uridine 5’-diphosphate, G-1-P glucose 1-phosphate, G-6-P glucose 6-phosphate, Gal-1-P galactose 1-phosphate, Ri-5-P ribulose 5-phosphate, R-5-P ribose 5-phosphate, PEP phosphoenolpyruvate, α-KG alpha-ketoglutarate, G-3-P glycerol 3-phosphate, 1,3-BPG glyceric acid 1,3-biphosphate, NADP nicotinamide adenine dinucleotide phosphate, FA fatty acids, GL glycerolipids, GP glycerophospholipids, SP sphingolipids, ST sterol lipids.

To elucidate the role of SNRNP200 in metabolic regulation through RNA splicing, we conducted untargeted metabolomic analysis on WT and SNRNP200-knockdown cells. Depletion of SNRNP200 led to significant reductions in metabolites essential for glycolysis and glutathione metabolism, including lactic acid, oxidized glutathione, UDPG, NAD (nicotinamide adenine dinucleotide), and SAM (Fig. 6a and Supplementary Table S4). These metabolites were particularly enriched in glycolytic subtypes (Fig. 1f), highlighting the pivotal role of SNRNP200 in these pathways. Additionally, the levels of specific lipids, such as diacylglycerol (DAG), phosphatidylethanolamine (PE), phosphatidylserine (PS), and phosphatidylcholine (PC), increased following SNRNP200 knockdown (Fig. 6b and Supplementary Table S5). Pathway-based differential abundance (DA) analysis confirmed alterations in metabolic pathways due to SNRNP200 depletion (Supplementary Fig. S9a).

We further explored whether the retention of introns in genes encoding key enzymes had a discernible impact on cellular metabolites. SNRNP200 knockdown led to intron retention in *ALDOA* (intron 9), *GAPDH* (introns 4 and 5), and *GSS* (intron 4), causing frameshift mutations and premature stop codons (Fig. 6c and Supplementary Fig. S9b). This resulted in reduced protein levels of ALDOA, GAPDH, and GSS (Fig. 6d). Clinically, U5 snRNP *SNRNP200* expression was positively correlated with *ALDOA*, *GAPDH*, and *GSS* in TNBC patient cohorts (Supplementary Fig. S9c). Metabolites in the glycolytic and glutathione pathways, such as lactate, glutamic acid, and glutathione, were significantly downregulated following SNRNP200 depletion (Fig. 6f). The extracellular acidification rate (ECAR) also decreased in SNRNP200-deficient cells (Fig. 6e). Additionally, introns within the genes encoding metabolic enzymes of lipid pathways, specifically diacylglycerol kinase (DGKZ) and phosphatidylserine decarboxylase (PISD), were retained after SNRNP200 knockdown. This retention might inhibit the transformation of metabolites, leading to the accumulation of PS and DAG (Supplementary Fig. S9d). These results indicate that SNRNP200 regulates the RNA splicing of specific metabolic enzyme-encoding genes, thereby influencing cellular metabolism.

To further examine whether the tumor-promoting function of SNRNP200 relies on its regulation of enzymatic activities and cellular metabolism, we conducted a rescue assay. Restoring ALDOA, *GAPDH*, or *GSS* expression in SNRNP200-knockdown cells partially restored lactic acid and glutathione levels (Supplementary Fig. S10a–c). Cell function assays revealed that restoring these enzymes partially rescued the proliferation inhibition caused by SNRNP200 knockdown (Supplementary Fig. S10d–f). Flow cytometry revealed a partial reduction in early- and late-stage apoptosis in SNRNP200-knockdown MPS2 cells upon enzyme restoration (Supplementary Fig. S10g). These results confirm that the tumor-promoting function of SNRNP200 is linked to its regulation of enzymatic activities and cellular metabolism.

### ASO-*Snrnp200* enhances immunotherapy efficacy in glycolytic MPS2 tumors

**Fig. 7:**
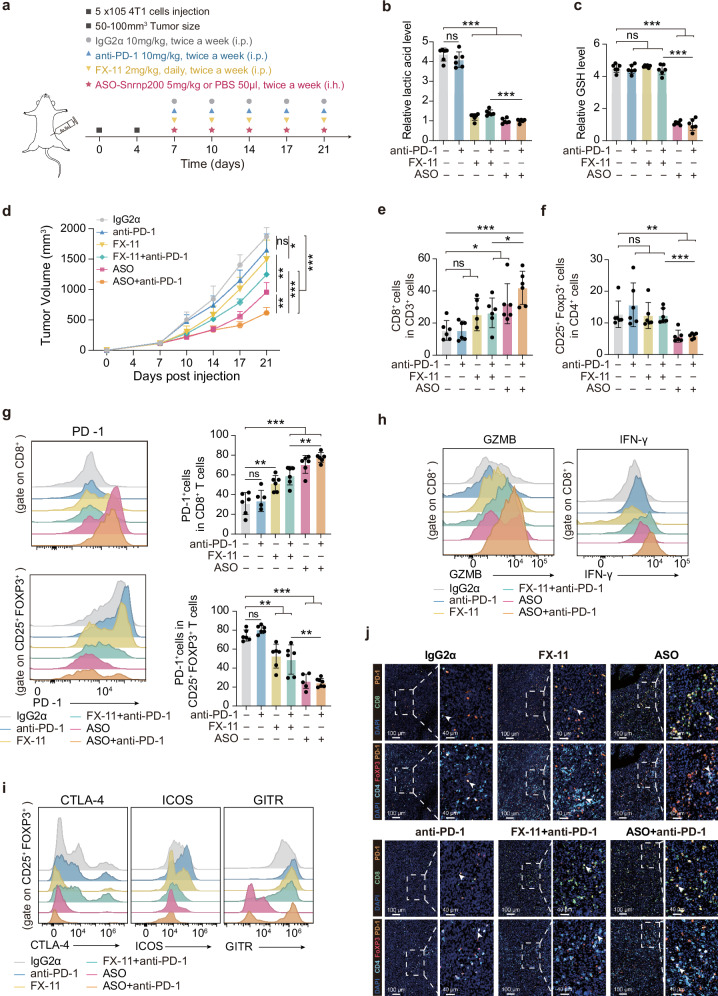
ASO-*Snrnp200* synergistic immunotherapy amplifies the antitumor response in glycolytic tumors. **a** Schematic outline showing *Snrnp200*-targeted ASO treatment or LDH inhibition combined with anti-PD-1 antibody treatment of tumors: 5 × 10^5^ 4T1 mouse breast cancer cells were subcutaneously injected into BALB/c mice. When the tumors reached 50–100 mm^3^, the mice were treated with ASO-*Snrnp200* (5 mg/kg subcutaneous injection, twice a week, *n* = 6 mice/group), FX-11 (2 mg/kg daily i.p. injection, daily), or PBS (50 µL, i.p. injection, daily) for 2 weeks combined with an isotype control or anti-PD-1 antibody (10 mg/kg, i.p. injection, twice a week). **b**, **c** Relative lactic acid (**b**) and GSH (**c**) levels in the six treatment groups. The data are presented as the mean ± SEM. **d** Tumor growth in different groups. The data are presented as the mean ± SEM. **e**, **f** Primary tumors from 4T1 model mice were harvested for flow cytometry to determine the percentages of CD8^+^ T cells among CD3^+^ T cells (**e**) and of Treg cells among CD4^+^ T cells (**f**). **g** The expression of PD-1 by CD8^+^ T cells (top) and Treg cells (bottom) in the tumor microenvironment was examined. Representative histograms and summary data are shown. The data are presented as the mean ± SEM. **h** Representative histogram plots showing GZMB^+^ and IFN-γ^+^ cells among CD8^+^ T cells. **i** Representative histogram plots showing CTLA4^+^, ICOS^+^, and GITR^+^ cells among Treg cells. **j** Representative multiplexed immunohistochemistry images of PD1^+^CD8^+^ T cells (top) and PD1^+^CD4^+^FOXP3^+^ T cells (bottom) in the TME. Scale bars, 100 μm or 40 μm. For **b**–**g**, the data were compared using Student’s *t*-test if the data in each group were normally distributed (*n* = 6 mice/group; *P < 0.05; **P < 0.01; ***P < 0.001; ns not significant, *P* > 0.05).

Extensive research has explored the intricate link between immunological responses and metabolic status. Recent studies have elucidated the pivotal role of regulatory T (Treg) cells, which function as metabolic conductors, in shaping immune responses via nutritional cues^26^. Glutathione has emerged as a crucial factor in preserving Treg functionality, whereas lactic acid in highly glycolytic tumor microenvironments promotes PD-1 expression in Treg cells, contributing to resistance to PD-1 blockade therapy and inhibiting tumor-infiltrating CD8^+^ T cells^27,28^. Here, we investigated whether SNRNP200 affects immune functions by modulating lactic acid and glutathione levels and whether ASO-*Snrnp200* treatment enhances immunotherapy sensitivity in glycolytic TNBC. In MPS2 tumor cells (4T1), ASO-*Snrnp200* reduced both the intracellular and extracellular lactic acid and glutathione levels in vitro (Supplementary Fig. 11a, b). Consistently, ASO-*Snrnp200* treatment significantly decreased Snrnp200 expression and lactic acid and glutathione levels in 4T1 xenograft tumors in vivo (Fig. 7a–c and Supplementary Fig. S11c). Compared with the LDH inhibitor FX-11 plus anti-PD-1, co-administration of ASO-*Snrnp200* and anti-PD-1 markedly inhibited tumor growth (Fig. 7d and Supplementary Fig. S11d, e). To further elucidate the immune response following treatment, we employed flow cytometry analysis (Supplementary Fig. S11f, g). The combined therapy yielded a pronounced immune response characterized by increased proportions of tumor-infiltrating CD8^+^ T cells (Fig. 7e), accompanied by elevated PD-1 expression within these cells (Fig. 7g). Moreover, CD8^+^ T cells in the combination therapy group presented increased IFN-γ and granzyme B production (Fig. 7h and Supplementary Fig. S12a, b). The combination therapy downregulated FOXP3 and PD-1 expression in Tregs (Fig. 7f, g) and reduced the expression of activation markers (CTLA-4, ICOS, and GITR; Fig. 7i and Supplementary Fig. S12c–e). mIHC staining of tumors for CD8^+^ T cells and Tregs consistently verified these observations (Fig. 7j). Our findings underscore the potential of ASO-*Snrnp200* to increase immunotherapy efficacy, highlighting its role in modulating tumor microenvironment dynamics, which are beneficial for therapeutic outcomes.

### Limited immunotherapeutic efficacy in TNBC patients with elevated SNRNP200 levels

**Fig. 8:**
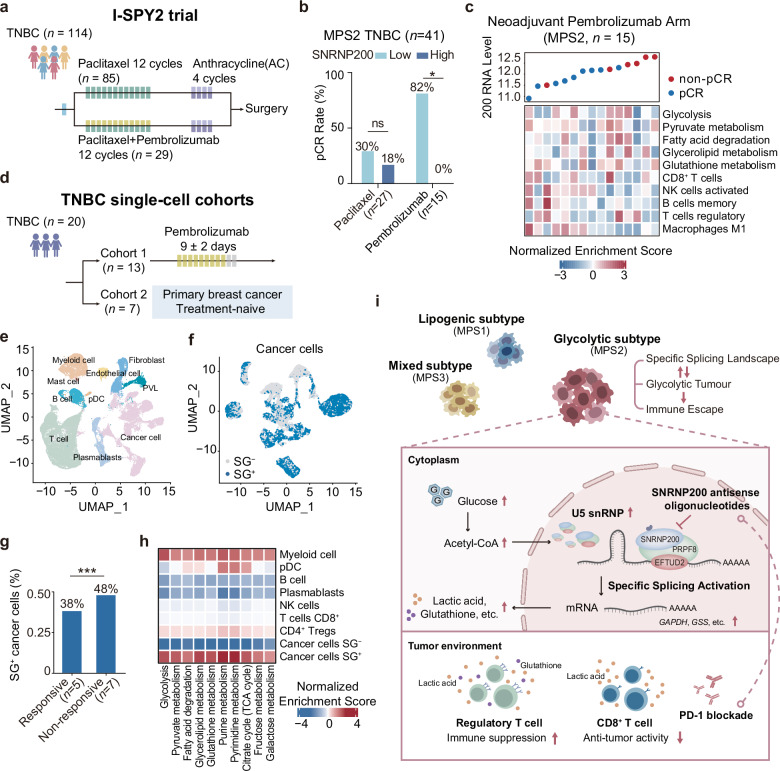
There is limited immunotherapeutic efficacy in TNBC patients with elevated SNRNP200 levels. **a** Overview of the I-SPY2 clinical trial (ClinicalTrials.gov: NCT01042379), which included 114 TNBC patients, with 29 receiving pembrolizumab plus chemotherapy and 85 receiving chemotherapy. **b** Bar chart illustrating SNRNP200 expression in 41 glycolytic TNBC patients with confirmed responses, categorized as pCR vs nonpCR. SNRNP200 expression was dichotomized by the median, and patient responses were stratified in both the immunotherapy and chemotherapy arms of the I-SPY2 clinical trial. **c** Expression of SNRNP200, normalized enrichment scores of MPS2-upregulated metabolic pathways, and proportions of representative infiltrating immune cells in the pembrolizumab group (MPS2, *n* = 15). **d** Overview of two scRNA-seq datasets, comprising a first cohort of 13 TNBC patients treated with pembrolizumab (BioKey study, ClinicalTrials.gov: NCT03197389) and a second cohort of 7 treatment-naïve TNBC patients (GSE176078). **e** Uniform manifold approximation and projection (UMAP) map of 70,190 cells color-coded for the indicated cell type. pDC plasmacytoid dendritic cell. PVLs perivascular-like cells. **f** UMAP map of 22,017 cancer cells grouped on the basis of AUCell values, with cells categorized as SG^+^ and SG^−^. **g** Proportion of SG^+^ cancer cells in patients who responded or did not respond to immunotherapy. **h** Heatmap depicting normalized enrichment scores of MPS2-upregulated metabolic pathways in the indicated cell types. **i** Schematic cartoon depicting the mechanism by which targeting *SNRNP200* promotes an antitumor immune response in glycolytic tumors. For **b**, **g**, the data were analyzed using the chi-squared test (*P < 0.05; ***P < 0.001; ns not significant, *P* > 0.05).

To assess the potential of SNRNP200 as a predictor of therapeutic response in TNBC patients receiving immunotherapy, we conducted our investigation within the I-SPY2 neoadjuvant platform trial (ClinicalTrials.gov: NCT01042379)^29^. The I-SPY2 trial focused on high-risk, early-stage breast cancer, with the primary endpoint being a pathological complete response (pCR). The control arm followed a standard-of-care regimen, including sequential weekly paclitaxel followed by doxorubicin/cyclophosphamide. Among the 114 TNBC patients enrolled, 29 received pembrolizumab plus chemotherapy, and 85 received chemotherapy (Fig. 8a). Intriguingly, patients treated with immunotherapy who displayed high SNRNP200 expression in their tumors exhibited a noteworthy reduction in the pCR rate — a phenomenon not observed in patients undergoing chemotherapy (Fig. 8b). Furthermore, TNBCs with higher SNRNP200 expression displayed increased glycolytic activity, enhanced glutathione metabolism, and elevated infiltration of CD8^+^ T cells (Fig. 8c).

To further investigate the predictive role of SNRNP200 in cancer cell states, we utilized two single-cell RNA sequencing (scRNA-seq) datasets (Fig. 8d). The first dataset included 13 TNBC patients treated with pembrolizumab (BioKey study, ClinicalTrials.gov: NCT03197389), whereas the second comprised 7 treatment-naïve TNBC patients (GSE176078)^30,31^. Following quality control, data integration, batch effect removal, and cell annotation, we identified 2,2017 cancer cells (Fig. 8e). We scored the expression levels of 42 spliceosome genes, including SNRNP200, upregulated in glycolytic TNBC at the single-cell level. The relative scores of these genes across individual cells enabled the identification of cells exhibiting significantly elevated RNA splicing activity. Two distinct peaks in the area under the curve (AUC) values were observed, with 12,873 cells demonstrating higher AUC values when the threshold was set to 0.16 (Supplementary Fig. S12f). Cancer cells above the 0.16 threshold were designated as spliceosome gene-positive (SG^+^) cells, whereas those below the threshold were categorized as spliceosome gene-negative (SG^–^) cells (Fig. 8f). Notably, we observed greater numbers of SG^+^ cancer cells and AUC values in patients who responded to immunotherapy (48% vs 38%; Fig. 8g and Supplementary Fig. S12g). Consistently, pathway enrichment analyses revealed elevated glucose and nucleic acid metabolism in SG^+^ cancer cells (Fig. 8h).

In summary, our research underscores the crucial role of SNRNP200 in glucose-driven metabolic dysregulation. Elevated glucose levels lead to acetylation at K1610, stabilizing SNRNP200 and enhancing gene splicing of metabolic enzymes, resulting in increased lactic acid and glutathione production. Targeting SNRNP200 with ASO therapy improves anti-PD-1 therapy efficacy by activating intratumoral CD8^+^ T cells and suppressing Treg cells. Our findings suggest that the integration of ASO-*SNRNP200* with immunotherapy is a promising option for the treatment of glycolytic TNBCs (Fig. 8i).

## Discussion

Here, our multiomics analysis elucidated the intricate relationship between RNA splicing and metabolic dysregulation. We found that the glycolytic subtype was hypersensitive to splicing inhibition and identified SNRNP200 as a pivotal factor orchestrating metabolic disturbances. Treatment with *SNRNP200* ASO impeded tumor metabolism and enhanced the efficacy of anti-PD-1 therapy for treating glycolytic TNBCs.

TNBC displays substantial metabolic reprogramming^32^. Although previous research revealed metabolic heterogeneity in TNBC, the precise underlying mechanisms remain elusive^4^. Over the past decade, studies have emphasized the role of RNA splicing in disrupting cell metabolism^33–35^. Nevertheless, a comprehensive understanding of splicing patterns in metabolic dysregulation within tumors is lacking. Our study revealed significant splicing deregulation in glycolytic TNBCs, particularly highlighting the correlation between U5 snRNP and elevated glucose and nucleic acid metabolism. We detected elevated levels of specific metabolites, including UDPG and SAM, which are specific to glycosyl and methyl transfer and are regulated by U5 snRNP, in glycolytic TNBC. While TNBC exhibits substantial variation in pathways related to lipid and carbohydrate metabolism^4,7^, our findings emphasize a predominant interaction between RNA splicing and carbohydrate metabolism, especially in highly glycolytic tumors. This association is supported by previous studies linking MYC-driven splicing deregulation to a glycolytic phenotype^17,36–38^, driven by the increased demand for glycolytic intermediates to support the de novo synthesis of nucleotides, lipids, and amino acids essential for cell proliferation^39,40^. This increased synthesis imposes an additional processing burden on cancer cells, augmenting their reliance on pre-mRNA splicing components, which is partly due to elevated pre-mRNA synthesis and heightened demands on the spliceosome^38,41,42^.

Our study further elucidated the critical role of U5 snRNPs, particularly SNRNP200, in mediating splicing dynamics that impact metabolic regulation in TNBC. Previous research has shown the importance of SNRNP200 in other cancers. For example, Katherine Knorr et al. linked SNRNP200 to CD32A expression and interferon-regulated genes in acute myeloid leukemia^43^. Similarly, Jiménez-Vacas JM et al. reported that SNRNP200 overexpression is correlated with prostate cancer aggressiveness^44^. However, its role in breast cancer, particularly TNBC, was previously unexplored. Moreover, the presence of intron-retaining transcripts associated with the active spliceosome (B^act^), remodeled by SNRNP200, was reported as a rapid adaptation mechanism for cancer cells under stress conditions^45,46^. Depletion of core U5 snRNP components disrupts transcription-coupled splicing of RNAs with weak 5’ splice sites^47^, leading to altered protein levels and downstream metabolite production, thereby exacerbating metabolic dysregulation. Specifically, changes in splicing mediated by SNRNP200 influence enzymes such as glycerol-3-phosphate acyltransferase 2 (GPAT2), an isozyme of GPAT that catalyzes glycerophospholipid synthesis. This results in increased production of lysophosphatidic acid (LPA), which has differential effects on glycolysis-related genes. This dual effect reflects the U5 snRNP’s differential influence on lipid vs glucose metabolism. Additionally, cancer-specific loss of isozyme diversity, exemplified by lower *GPAT2* RNA levels in TNBC than in *GPAT1*^48^, suggests potential mitigation of LPA production upon SNRNP200 inhibition. The complexity of RNA splicing and metabolic reprogramming in cancer biology is further highlighted by the regulation of glycolysis-related genes through the splicing isoforms of pyruvate kinase muscle (PKM), such as PKM1 and PKM2^13^. PKM1 is prevalent in most adult tissues, whereas PKM2 is predominant in tumor tissues, supporting biosynthesis and proliferation through metabolic reprogramming and signaling pathway modulation^49^. Additionally, the HIF-1α antisense long noncoding RNA drives the PKM2/PHD3 complex into the nucleus, establishing a positive feedback loop that enhances glycolysis in cancer cells^50^.

Metabolites play crucial roles beyond traditional metabolic functions in cancer biology. For example, elevated glucose directly binds to DDX21, disrupting its dimerization and affecting the splicing of critical pro-differentiation genes^51^. Furthermore, metabolites derived from glycolysis and the Krebs cycle, such as lactate and acetyl-CoA, facilitate posttranslational modifications (PTMs) of proteins^52,53^, enabling rapid adjustments to the cellular energy status and the modulation of reaction rates to promptly respond to environmental changes^54,55^. Our research specifically highlights how elevated glucose levels in glycolytic TNBCs induce the acetylation of SNRNP200 at lysine 1610, stabilizing it against deacetylation and ubiquitylation processes and thereby protecting SNRNP200 from proteasomal degradation. These findings underscore the concept that metabolite-driven PTMs influence RNA splicing dynamics, revealing the unconventional role of metabolites in cancer biology.

Our study investigated the profound impact of metabolite alterations on cellular functions, particularly in the context of TNBC. Consistent with prior research^56,57^, we revealed that TNBC, particularly the MPS2 subtype, displays distinct RNA splicing patterns in comparison with luminal subtypes or HER2-enriched breast cancer, featuring more pronounced splicing dysregulation^57,58^. These splicing anomalies are predominantly linked to dysregulated core spliceosome components^15^. Emerging evidence suggests that mutations in core spliceosome components do not generally affect splicing but preferentially lead to changes in specific AS events^47,59^. This phenomenon could be partially explained by the order and duration of their recruitment to the spliceosome^16^. The pivotal role of splicing in tumorigenesis has led to increasing interest in developing various approaches for cancer therapy in both preclinical and clinical settings. One noteworthy broad-spectrum spliceosome inhibitor is isoginkgetin, which disrupts the recruitment of the U4/U6.U5 tri-snRNP, leading to stalling at the prespliceosomal A complex and inducing tumor cell death^60,61^. Furthermore, our study revealed significant metabolic heterogeneity within TNBC subtypes, particularly between the MPS1 and MPS2 subtypes. MPS1 tumors predominantly exhibit areas positive for fatty acid synthase (FASN) with partial LDH positivity, whereas MPS2 tumors show LDH-positive areas alongside partial FASN positivity. Importantly, core U5 snRNP proteins, SNRNP200, EFTUD2, and PRPF8, are notably upregulated in MPS2 TNBCs with LDH-positive areas, highlighting distinct metabolic profiles and vulnerabilities among TNBC subtypes. Our previous research also underscored the critical roles of core spliceosome components in cancer biology. For example, PHF5A, a core component of U2 snRNP, influences apoptotic signaling in TNBC^19^. Additionally, our work on SNRPC, a U1 snRNP subunit, highlighted its regulatory role in TNBC progression. These studies collectively suggest that targeting specific core spliceosome genes can induce selective cell death in TNBC cells without affecting normal breast epithelial cells, highlighting RNA splicing as a promising therapeutic avenue^20^. In our investigation, SNRNP200 emerged as a key player in glycolytic TNBCs, with *SNRNP200* ASO emerging as a promising therapeutic option for this subtype. The susceptibility of glycolytic TNBCs to splicing inhibition underscores that the *SNRNP200* ASO is a highly selective inducer of tumor cell death, offering potential advantages over traditional metabolic drugs by minimizing adverse effects on non-cancer stromal and immune cells. Furthermore, based on the observed reduction in lactate and glutathione production following *SNRNP200* ASO treatment, we propose that combining ASO-*SNRNP200* with immunotherapy could enhance this glycolytic subset.

In conclusion, our study advances the understanding of RNA splicing and its regulatory role in metabolic reprogramming in TNBC. By revealing the intricate connections among SNRNP200-mediated splicing dynamics, metabolite-driven PTMs, and metabolic profiles in TNBC subtypes, we provide new insights into cancer biology and therapeutic opportunities. Future research should focus on validating these findings in clinical settings and exploring additional spliceosome components and their interactions with metabolic pathways to refine targeted therapies for TNBC patients.

## Materials and methods

### Patient cohorts

Our study comprised two patient cohorts with TNBC from FUSCC: the FUSCC-TNBC and the FUSCC-PCMC cohorts. The FUSCC-TNBC cohort included 465 TNBC patients (100% female, average age = 53 ± 11 years). Among these, RNA-seq data were available for 360 patients, proteomic data for 90 patients, and metabolomic data for 72 patients. The FUSCC-PCMC cohort consisted of 179 samples with transcriptomic data collected from breast cancer patients treated at the Department of Breast Surgery at FUSCC between July 2023 and January 2024. The enrollment criteria for this cohort were as follows: (1) female patients diagnosed with unilateral invasive ductal carcinoma with an ER, PR, or HER2 phenotype; (2) central pathologic examination of tumor specimens performed by the Department of Pathology at FUSCC, with ER, PR, and HER2 status independently confirmed by two experienced pathologists via IHC and in situ hybridization. We used < 1% positively stained cells as the cutoff for ER/PR negativity in IHC according to the American Society of Clinical Oncology/College of American Pathologists guidelines^62,63^; and (3) the availability of sufficient fresh tumor tissue for further research. All tissue samples were obtained following approval by the FUSCC Ethics Committee. Each patient provided written informed consent for data and tissue use. The TCGA cohort (a total of 1096 patients, 98.9% female and 1.1% male, average age = 58 ± 13 years; including 161 patients with TNBC with RNA-seq data, 100% female, average age = 55 ± 12 years) were analyzed for external validation.

### Human and mouse TNBC cell lines

The human breast cancer cell lines MCF-7, BT474, AU565, HCC1954, SK-BR-3, BT-20, BT-549, HCC1143, Hs578T, MDA-MB-231, and MDA-MB-468; the human breast epithelial cell line MCF10A and the human embryonic kidney cell line HEK293T were obtained from the American Type Culture Collection (ATCC). All TNBC breast cancer cell lines were classified into TNBC subtypes. The 4T1 mouse TNBC cell line was obtained from Y. Kang’s laboratory (Princeton University, USA). All the above cells were identified via cell line DNA profiling (short tandem repeat, STR) and passed routine cell line quality control tests (e.g., morphology, cell viability, and mycoplasma). MCF10A cells were maintained in DMEM/F12 (BasalMedia, L310KJ) supplemented with 5% horse serum (Gibco, SR0035), 1% penicillin-streptomycin (BasalMedia, S110JV), 10 μg/mL insulin (BasalMedia, S450J7), 100 ng/mL cholera toxin (Sigma-Aldrich, 227036), 0.5 μg/mL hydrocortisone (Sigma-Aldrich, 3867) and 20 ng/mL EGF (PeproTech, 100-47). The other cell lines were cultured according to standard conditions. Other cell lines were maintained in high-glucose DMEM supplemented with 10% fetal bovine serum (Gibco, 16000044) and 1% penicillin-streptomycin. All the cell lines were passaged fewer than 30 times and cultured in a humidified incubator containing 5% CO2 at 37 °C. TSA (10 μM, MedChemExpress, HY-15144) and NAM (5 mM, MedChemExpress, HY-B0150) were added to the culture media 16 h and 6 h before harvest, respectively. Glucose-free medium was prepared with DMEM (Gibco, 11966) supplemented with glucose (Sigma-Aldrich, G7528) as indicated.

### Xenograft models

All animal experiments were performed according to protocols approved by the Research Ethical Committee of FUSCC. Six- to seven-week-old female BALB/c mice were used in this study. The mice were housed under a 12-h light/dark cycle at ambient temperature (20–22 °C) and humidity (60 ± 10%) with free access to standard rodent diet and water in individually ventilated cages in a specific pathogen-free facility to monitor health status via culture, serum and microscopic examination.

### Dimensionality reduction analysis

PCA determines the variable combinations that contribute the most to data variance and is accomplished via the singular value decomposition (SVD) method given by the prcomp function from the R package stats (version 3.4.1)^64^. The overall contribution of each variable (splicing event or gene) to the data variance along specified main components was calculated via fviz_contrib from factoextra (version 1.0.5). *t*-SNE was used for dimensionality reduction and visualization of high-dimensional data via the Rtsne (version 0.15) package^65^.

### WGCNA

First, we calculated the Pearson correlation coefficient between all gene pairs across samples. Next, the correlation coefficients were transformed into an adjacency matrix with a soft threshold of β = 4. The matrix was then transformed into a topological matrix with the topological overlap measure (TOM), which describes the degree of association between genes. A 1-TOM was used as the distance to cluster the genes, after which a dynamic pruning tree was constructed, and the 84 MPS2-related modules were identified by setting the merging threshold function at 0.25. We computed the eigengene for each module, defined as the first principal component of the module representing the overall expression level of the module, as well as module membership (MM). We quantified associations of individual genes with our clinical variables by defining gene significance as (the absolute value of) the correlation between the gene and the trait. For each module, we also defined a quantitative measure of MM as the correlation of the module eigengene and the gene expression profile. This allowed us to quantify the similarity of all genes on the array to every module. Based on the genes of significantly related modules (yellow modules: cor = 0.87, *P* < 0.001), two genes with weights > 0.02 were used to construct the network.

### Correlations between core spliceosome genes and metabolic pathways

GSVA, performed via the R package GSVA (version 1.36.2), was utilized to calculate the enrichment score for each pathway in every sample or cell via log_2_-transformed FPKM^66^. The correlation between log_2_ expression levels of 42 genes coding for core spliceosome components and metabolic pathways based on the standard error of the enrichment score was tested via Spearman’s rho by cor.test from stats (Version 4_4.0.3).

### Western blot analysis

Proteins were extracted via Tissue Protein Extraction Reagent (T-PER, ThermoFisher Scientific, 78510) in combination with proteinase and phosphatase inhibitors (Sigma-Aldrich, 5892970001). The protein concentration was then determined via BCA reagent (Solarbio, PC0020), and the sample was boiled for 10 min in an SDS-PAGE loading buffer. Equal concentrations of proteins were electrophoresed on SDS-PAGE gels and transferred to PVDF membranes. The membranes were blocked with 5% skim milk for 60 min before being probed with primary antibodies for 12–16 h at 4 °C. After thorough washing with TBST, the membranes were incubated for 1 h with a goat anti-rabbit antibody (Cell Signaling Technology, 7074) or a goat anti-mouse antibody (Cell Signaling Technology, 7076) at room temperature. The targeted protein signals were identified via an enhanced chemiluminescence substrate (Pierce Biotechnology) and visualized utilizing Image Lab Software (Bio-Rad). Triplicates of each experiment were run. A list of the primary antibodies used for immunoblotting is provided in Supplementary Table S8.

### SNRNP200 knockdown

For CRISPR/Cas9-mediated knockdown of human SNRNP200, first, cells transfected with lentivirus containing lentiCas9-Blast (Addgene, 52962) were selected with blasticidin (InvivoGen, ant-bl-10p) for at least 2 weeks. Next, the sgRNA lentivirus was introduced into Cas9 cells. Forty-eight hours after lentiviral sgRNA transfection, stably integrated cells were selected with 1–2 µg/mL puromycin (InvivoGen, ant-pr-1) for 7 days. These cells were used for subsequent assays (RNA-seq, qPCR, Western blot, etc.). The knockdown efficiency was confirmed by western blot analyses. The *SNRNP200*-sgRNA sequences used were as follows: *SNRNP200*-sg1: ACCCGCCGGGATGAACCCAC; and *SNRNP200*-sg2: TAGGCGAAGAAAGCGTGATG.

### RNA treatment in endogenous IP assays

For RNA treatment in the endogenous IP assay, 3 × 10^7^ breast cancer cells were collected and lysed with predetermined IP buffer (50 mM Tris-HCl pH 8.0, 100 mM NaCl, 1 mM EDTA, and 1% NP-40). After a 1-h rotation at 4 °C, the lysates were subjected to centrifugation at 12,000 rpm for 5 min at 4 °C. A 10 μL aliquot of each sample was set aside as input for RNA extraction, whereas another 10 μL was designated as input for western blot analysis. The lysates were then categorized into three distinct groups: the IgG, RNase A^−^ (no RNase A treatment), and RNase A^+^ (with RNase A treatment, 100 μg/mL, ThermoFisher Scientific, R1253) groups. Afterward, 30 μL of lysate was removed from both groups for RNA extraction. The remaining samples were incubated with the following antibodies for the IP assay: anti-IgG (Cell Signaling Technology, 2729) and anti-SNRNP200 (Abcam, ab176715). After rotating overnight at 4 °C, the samples were centrifuged to precipitate the beads. After washing with IP buffer five times, the beads were boiled for 10 min with 40 µL of 1× loading buffer for each sample. Finally, the samples were analyzed by western blot analysis as previously described. After cDNA synthesis, the U4, U5, and U6 snRNA levels in the input, RNase A^−^ and RNase A^+^ groups were detected using RT-qPCR. The relative U4, U5, and U6 snRNA levels of the RNase A^−^ and RNase A^+^ groups were normalized to those of the input group via the 2^−^^△CT^ method.

### In vitro cell viability assays

For the cell proliferation assay, cells were seeded in quintuplicate in a 96-well plate (1 × 10^3^ cells/well) and cultivated in an incubator containing 5% CO_2_ at 37 °C. Cell viability was measured via a cell counting kit‐8 (CCK8; MedChemExpress, HY-K0301), and the highest and lowest values were excluded. For the EdU incorporation assay, an EdU kit (RiboBio, C10310) was used to detect the proliferation of breast cancer cells. In brief, treated cells (100 μL of a 2 × 10^4^ cells/mL suspension) were seeded into 96-well plates, and 50 mM EdU was added at 37 °C for a 2-h incubation. After fixation with 4% paraformaldehyde and permeabilization with 1% Triton X-100, 100 μL of click reaction cocktail was added to the treated cells, and the cells were then stained with Hoechst 33342. Images of the immunostained sections were captured and quantitatively evaluated with a fluorescence microscope (Olympus). For the colony formation assay, cells were seeded in triplicate in a 6-well plate (1 × 10^3^ cells/well) and cultivated under normal conditions. After two weeks, the colonies were fixed in methanol and stained with 0.05% crystal violet for 30 min. Colonies with more than 50 cells were counted.

### Flow cytometry measurement of cell apoptosis

The cells of interest were fixed and stained with Annexin V binding buffer (BD Biosciences, 556454) according to the manufacturer’s instructions. The apoptotic cells were then identified and analyzed with FlowJo software.

### IP-LC-MS

HEK293T cells transfected with the p*CMV6-SNRNP200*-Myc plasmid were lysed and immunoprecipitated with Protein A/G magnetic beads (Bimake, B23202) in IP buffer. The eluted FLAG peptide was resolved by SDS-PAGE and Coomassie blue staining. Lysates from HEK293T cells transfected with control pCMV6-Entry (Origene, PS100001) served as controls. Protein bands specific to Myc-SNRNP200-transfected cells were digested with trypsin for sequencing by MS analysis (Novogene Bioinformatics Institute, Beijing, China). Protein identification was performed via Proteome Discoverer 2.2 (PD2.2, ThermoFisher Scientific) in the human RefSeq protein database (National Center for Biotechnology Information).

### In vivo ubiquitylation assays

For the analysis of SNRNP200 ubiquitylation, HEK293T cells were transfected with plasmids, including pCMV6-*SNRNP200*-Myc, pCMV6-*SNRNP200*-K1610Q-Myc, pCMV6-*SNRNP200*-K1610R-Myc, pRK5-HA-*Ubiquitin*-WT (Addgene, 17608), pRK5-HA-*Ubiquitin*-K63 (Addgene, 17606), pRK5-HA-*Ubiquitin*-K48 (Addgene, 17605), pCMV-*RNF123*-FLAG, pCMV-*HDAC5*-FLAG, and pcdna3.4-*KAT2B*-FLAG, as indicated. Prior to being harvested for IP experiments, HEK293T cells were pretreated with 10 μM MG132 (MedChemExpress, HY-13259) for 10–12 h to inhibit the proteasome pathway. After 48 h of transfection, the cells were lysed and subsequently incubated with anti-FLAG (Cell Signaling Technology, 14793) or anti-Myc-Tag (Cell Signaling Technology, 2278) antibodies overnight at 4 °C, followed by further incubation with protein A/G magnetic beads (Bimake, B23202) for an additional 8 h at 4 °C. After five washes, ubiquitinated SNRNP200 was detected by immunoblotting with an anti-HA antibody (Abcam, ab9110). To detect the endogenous ubiquitylation of SNRNP200, BT-549 cells were transfected with pCMV6-*SNRNP200*-Myc plasmids along with pRK5-HA-*Ubiquitin*-WT (Addgene, 17608), and subjected to si*RNF123* or control treatment. Additionally, the cells were exposed to the proteasome inhibitor MG132 (10 μM, MedChemExpress, HY-13259) for 10 h. The cellular proteins were then subjected to IP using an anti-SNRNP200 antibody (Abcam, ab176715), followed by immunoblotting with an anti-HA antibody (Abcam, ab9110) for analysis.

### In vivo acetylation assays

For SNRNP200 acetylation analysis, HEK293T cells were transfected with the indicated plasmids. and pretreated with or without TSA (10 μM, MedChemExpress, HY-15144) for 16 h or NAM (5 mM, MedChemExpress, HY-B0150) for 6 h before being harvested for IP experiments. The level of acetylated SNRNP200 was determined by immunoblotting with an anti-acetyl-lysine antibody (Abcam, ab190479).

### siRNAs and transfection

Specific siRNAs targeting *PCAF* and *RNF123*, along with corresponding negative control siRNAs (siNCs), were obtained from Tsingke Biotechnology (Shanghai, China). The siRNA duplexes were introduced into cells via Lipofectamine 2000 transfection reagents (Invitrogen, 11668019), according to the manufacturer’s guidelines. The knockdown efficiency was assessed through immunoblotting analysis at 48 h posttransfection. The siRNA sequences are listed in the Supplementary Table S6.

### Acetyl-CoA measurement

After 16 h of exposure to glucose at various concentrations (0 mM, 2.5 mM, 7.5 mM, 12.5 mM, and 25 mM), 1 × 10^7^ BT-549 cells were rapidly frozen in liquid nitrogen (liquid N_2_) and then pulverized. Acetyl-CoA content assessment was conducted following the manufacturer’s guidelines (Sigma Aldrich, MAK039) using a fluorescence assay. The fluorescence intensity was measured (λ_ex_ = 535/λ_em_ = 587 nm) in black, 96-well flat-bottom plates with clear bottoms. Standard curves were generated using 0.02 mM acetyl-CoA standard solutions with volumes of 0 μL, 10 μL, 20 μL, 30 μL, 40 μL, and 50 μL.

### Splicing assays

Total RNA was extracted from the cells using TRIzol (Life Technologies, 15596018), and reverse transcription was carried out to generate complementary DNA (cDNA) using the HiScript III 1st Strand cDNA Synthesis Kit (Vazyme, R312). Primers were designed using SnapGene software (version 7.0) to amplify transcripts near alternatively spliced exons. For subsequent DNA gel electrophoresis separation, real-time PCR was performed using 2× Phanta Max Master Mix (Vazyme, #P515), while qPCR was carried out with SYBR qPCR Master Mix (Vazyme, #Q311). In this work, the relative expression level of each RNA was quantified using the 2^−ΔΔCT^ method, with ACTB serving as the internal standard. For the RI analyses, the cDNA samples were subjected to qPCR analyses with primers designed for the retained introns (In) and the adjacent exons (All). The percentage of transcripts with RIs was determined using the formula PSI = 2^CT(All) ^^−^ ^CT(In)^. Each experiment was conducted in triplicate. The primers used for the RT-PCR and qPCR analyses are summarized in Supplementary Table S7.

### Analysis of splice site and GC content

Using the MaxEntScan algorithm, the strength of splice sites was computed. A standard collection of background sequences was used to perform motif enrichment analysis with the R program ggseqlogo (version 0.1). To account for splicing signals, GC contents in introns versus neighboring exons were compared by deleting 20 nucleotides from either end of the introns and 3 nucleotides from the exons.

### RNA-seq

RNA extraction was completed as described previously. RNA-seq library construction was performed using the VAHTS mRNA-seq V2 Library Prep Kit for Illumina (Vazyme, NR612) according to the manufacturer’s instructions, followed by double-ended 150 nt sequencing using the Illumina NovaSeq 6000 platform. The FASTQ data were mapped to the human genome (hg38) using TopHat2, and index generation was performed using SAMtools and Picard.

### Metabolite extraction

MDA-MB-231 cells were frozen in liquid nitrogen until extraction. One milliliter of extraction solution (acetonitrile:methanol:water = 2:2:1) containing 2 mg/mL L-2-chlorophenylalanine as an internal standard was added to the samples. After 30 s of vortexing, the samples were homogenized at 40 Hz for 4 min and sonicated for 10 min in an ice-water bath. The homogenization and sonication cycles were repeated three times. The samples were subsequently incubated at −40 °C for 1 h and centrifuged at 10,000 rpm for 15 min at 4 °C. A total of 800 mL of the supernatant was transferred to a fresh tube and dried in a vacuum concentrator at 37 °C. The dried samples were reconstituted in 200 mL of 50% acetonitrile by sonication on ice for 10 min. The constitution was then centrifuged at 13,000 rpm for 15 min at 4 °C, and 75 mL of the supernatant was transferred to a fresh glass vial for LC/MS analysis.

### LC-MS/MS analysis

Ultrahigh-performance liquid chromatography (UHPLC) separation was performed via an Agilent 1290 Infinity UHPLC System with a UPLC BEH amide column. The mobile phase consisted of a mixture of 25 mmol/L ammonium acetate, 25 mmol/L ammonia hydroxide in water (pH = 9.75) (A), and acetonitrile (B). The column temperature was 25 °C, the autosampler temperature was 4 °C, and the injection volume was 2 mL (pos) or 2 mL (neg).

A 6550 QTOF mass spectrometer (Agilent Technologies) was used for full-scan MS1 data acquisition in the 60–1200 Da scan range. The ESI source conditions included a gas temperature of 250 °C, a gas flow of 16 L/min, a sheath gas temperature of 350 °C, a sheath gas flow of 12 L/min, a nebulizer at 20 psi, a fragmentor at 175 V, and a capillary voltage of 3000 V.

For MS/MS spectra, a Triple TOF 6600 mass spectrometer (AB Sciex) was employed in information-dependent acquisition (IDA) mode with Analyst TF version 1.7 (AB Sciex). The 12 most intense precursor ions with intensities above 100 were selected for MS/MS analysis at a collision energy (CE) of 30 eV. The cycle time was 0.56 s. The ESI source conditions included gas 1 at 60 psi, gas 2 at 60 psi, the curtain gas at 35 psi, the source temperature at 600 °C, the declustering potential at 60 V, and the ion spray voltage floating (ISVF) at 5000 V (positive) or −4000 V (negative).

### DA score

The DA score reflects a pathway’s tendency to exhibit higher metabolite levels than those in the control group. It is determined by initially subjecting all pathway metabolites to a nonparametric DA test (Mann–Whitney *U*-tests with Benjamini–Hochberg correction). The DA score was calculated based on the significantly increased or decreased metabolite levels, and the DA score was defined as:

$${\rm{DA}}=\frac{{\rm{No}}:{\rm{of}}\; {\rm{metabolites}}\; {\rm{increased}}-{\rm{No}}:{\rm{of}}\; {\rm{metabolites}}\; {\rm{decreased}}}{{\rm{No}}:{\rm{of}}\; {\rm{measured}}\; {\rm{metabolites}}\; {\rm{in}}\; {\rm{pathway}}}$$

Thus, the DA score varies from −1 to 1. A score of −1 indicates that all metabolites in a pathway decreased in abundance, whereas a score of 1 indicates that all metabolites increased.

### ECAR assays

The ECAR of the MDA-MB-231 cells was quantified using a Seahorse XFe96 Extracellular Flux Analyzer (Agilent Technologies). MDA-MB-231 cells were seeded at 10,000 cells per well in a 96-well XF cell culture microplate (Agilent Technologies) with the corresponding growth medium and incubated overnight in a 5% CO2 incubator. Approximately 1 h before the Seahorse Analyzer readings, the cells were washed and incubated in serum-free, bicarbonate-free XF base medium in a non-CO2 incubator. An XF Glycolysis Stress Test Kit (Agilent Technologies, 103020-100) was used to detect the ECAR according to the manufacturer’s instructions. The ECAR was determined after 12 cycles (3 min of mixing and 3 min of mixing). The baseline measurements were the average of the last three readings before oligomycin addition (1 mM final concentration).

### ASO delivery

All ASOs were designed and synthesized by RiboBio, China. The oligonucleotide concentration was determined with a Nanodrop spectrophotometer. For in vitro ASO delivery, 1 × 10^5^ 4T1 mouse breast cancer cells were seeded in six-well plates and transfected with ASOs at various concentrations (0 nM, 10 nM, 20 nM, 30 nM, 50 nM, and 100 nM) with 5 μL of Lipofectamine 3000. For viability assays, the protein was isolated after 48 h for protein knockdown assessment. For in vivo ASO delivery, ASOs were delivered by subcutaneous injection at a dose of 5 mg/kg. PBS was injected as the control.

### In vivo mouse studies

Six- to seven-week-old female BALB/c mice were obtained from Shanghai Jihui Laboratory Animal Care Co., Ltd. and maintained under pathogen-free conditions. All animal experiments were performed according to protocols approved by the Research Ethical Committee of FUSCC. A total of 5 × 10^5^ 4T1 mouse breast cancer cells were injected subcutaneously into the mammary fat pad region of each mouse. Tumor size was measured twice weekly using a caliper. For the *Snrnp200*-targeted ASO monotherapy assay for the 4T1 cell-derived xenograft model, the mice were divided into two groups: (1) the vehicle group (PBS, 50 µL, subcutaneous injection twice a week) and (2) the ASO-*Snrnp200* group (5 mg/kg, subcutaneous injection twice a week). For the combination therapy assay, the mice were injected with 4T1 cells and randomly divided into six groups: (1) vehicle (50 µL of PBS, injected intraperitoneally daily) plus isotype (IgG2a, 10 mg/kg injected intraperitoneally twice a week, Bio X Cell, BE0089); (2) vehicle (50 µL of PBS, injected i.p. daily) plus anti-PD-1 (RMP1-14, 10 mg/kg injected i.p. twice a week, Bio X Cell, BE0146); (3) anti-LDH (FX-11, 2 mg/kg injected i.p. daily, MedChemExpress, HY-16214) plus isotype (IgG2a, 10 mg/kg injected i.p. twice a week); (4) anti-LDH (FX-11, 2 mg/kg injected i.p. daily) plus anti-PD-1 (RMP1-14, 10 mg/kg injected i.p. twice a week); (5) ASO-*Snrnp200* (5 mg/kg, subcutaneous injection twice a week) plus isotype (IgG2a, 10 mg/kg injected i.p.twice a week); and (6) ASO-*Snrnp200* (5 mg/kg, subcutaneous injection twice). The tumor volume in mm^3^ was calculated using the formula: tumor volume = 0.5 × L × W^2^, where L is the longest dimension and W is the perpendicular dimension.

### Flow cytometry analysis

For flow cytometry analysis of in vivo experiments, mouse tumors were quickly excised and then mechanically dissociated with scissors in sterile PBS. Tumor fragments were digested in serum-free RPMI + 1 mg/mL collagenase D (Roche, 11088866001) + 1 mg/mL collagenase I (Sigma-Aldrich, C0130) + 20 mg/mL DNase I (Roche, 10104159001) for 30–60 min at 37 °C with rotation to promote dissociation. Single-cell suspensions were passed through a 70 mM cell strainer. The red blood cells in the tumor samples were then lysed with red blood cell lysis buffer (eBioscience, 00-4300-54) for 5 min at room temperature. Lysis reactions were quenched by the addition of 20 mL of PBS, and the samples were subsequently centrifuged at 300× *g* for 5 min at 4 °C. The cells were then washed in D-PBS and stained with fluorescently labeled antibodies against the following surface proteins at a 1:100 dilution in Cell Staining Buffer (BioLegend, 420201) for 30 min at 4 °C: PE-conjugated anti-mouse CD45 (ThermoFisher Scientific, 12-0451-82), CD3e (ThermoFisher Scientific, 17-0032-82), CD4 (BD Biosciences, 550954), CD25 (BD Biosciences, 553071), CD8a (ThermoFisher Scientific, 11-0081-82), CTLA-4 (BD Biosciences, 553720), PD-1 (BD Biosciences, 562523), and GITR (BD Biosciences, 558119). For all the intracellular protein analyses, the cells were then fixed and permeabilized by using fixation buffer (BioLegend, 420801) and intracellular staining permeabilization wash buffer (BioLegend, 421002) according to the manufacturer’s instructions. Permeabilized cells were then incubated with fluorescently labeled antibodies against FOXP3 (BD Biosciences, 560401), ICOS (BD Biosciences, 552146), granzyme B (BioLegend, 372214), and IFN-γ (BioLegend, 505850) for 30 min at 4 °C. A CytoFLEX S flow cytometer (Beckman Coulter) was used for flow cytometry data acquisition, and the data were analyzed with FlowJo software (version 10.5.3).

### mIHC analysis

As described previously, antibodies against SNRNP200 (Abcam, ab241589), EFTUD2 (Abcam, ab188327), PRPF8 (Abcam, ab79237), FASN (Abcam, ab128870), LDH (Abcam, ab52488), FOXP3 (Servicebio, GB112325), CD4 (Servicebio, GB15064), PD-1 (Cell Signaling Technology, 84651), and CD8a (Cell Signaling Technology, 98941) were used following the manufacturer’s instructions (Panovue, 0060000050). In brief, tissue sections were rehydrated in ethanol and deparaffinized in xylene. After a 15-min microwave antigen retrieval step in hot citric acid buffer (pH 6.0), endogenous peroxidase activity was quenched with 3% H_2_O_2_ for 10 min, and nonspecific binding sites were blocked with goat serum for 10 min. Following incubation with primary antibodies in a humidified room temperature environment for 1 h, the samples were treated with secondary horseradish peroxidase-conjugated polymers. To visualize each target, fluorescein tyramide signal amplification staining (1:100) was applied. An antigen retrieval step using microwave heating with citric acid buffer (pH 6.0) was performed before proceeding to the next stage to remove excess antibodies. Finally, antifade mounting media was used to cover the sections, and DAPI (Sigma-Aldrich, D5942) was utilized to visualize the cell nuclei. Whole-slide scanning fluorescence images were scanned via an Olympus VS200 and analyzed via OlyVia software (version 3.4.1). A list of the primary antibodies is provided in Supplementary Table S8.

### Lactic acid and glutathione content assays

For the lactic acid content assay of in vivo experiments, mouse tumors were detected with a lactic acid content detection kit (Solarbio, BC2235) according to the manufacturer’s instructions. For the glutathione content assay, the tumors were frozen in liquid nitrogen. GSH and GSSG assays were performed, and the GSH/GSSG ratio was calculated using a GSH/GSSG detection assay (Beyotime, S0053) according to the manufacturer’s instructions.

### CIBERSORT

The “Cell type Identification by Estimating Relative Subsets of RNA Transcripts (CIBERSORT)” calculated with the “kappa” function in R was used to calculate the abundance of 22 types of immune cell subsets in each sample^67^.

### AUCell

The AUCell R package (version 1.24.0) was utilized to assess RNA splicing activity in individual breast cancer cells. Initially, a set of 42 spliceosome genes upregulated in glycolytic TNBCs was used as input to derive a spliceosome gene score. The AUC was used to estimate the proportion of highly expressed genes within the set for each cell. Cells expressing a greater number of genes from this set demonstrated elevated AUC values. The ‘AUCell_exploreThresholds’ function determines the threshold for defining the active state of the current gene set. Then, UMAP embedding for cell clustering was color-coded based on the AUC of each cell.

### Statistics and reproducibility

Statistical significance was assessed using various tests, including the unpaired Student’s *t*-test, chi-square test, Mann‒Whitney test, and Kruskal‒Wallis test when applicable. Prior to comparisons, the normality of distributions was evaluated using the Shapiro‒Wilk test. The data are presented as the mean ± SEM of at least three independent experiments. Correlation matrices were generated using Pearson’s or Spearman’s correlation. All in vitro cell-based experiments were independently repeated three times in triplicate. The detailed *P* values and sample sizes can be found in the main and supplementary figure legends. Two-sided *P* values less than 0.05 were considered to indicate statistical significance. Statistical analyses were conducted using R software (http://www.R-project.org, version 3.5.2) or GraphPad Prism software (version 8.0).

## Supplementary information

Supplementary information

Supplementary Table

## Data Availability

The results shown here are in part based upon data generated from the TCGA cohorts and are available in a public repository from https://portal.gdc.cancer.gov/. The transcriptomic data from pretreatment tumors of the I-SPY2 cohort are also available at the Gene Expression Omnibus (GEO: GSE196093). For the scRNA-seq data, the BioKey study provides information available at https://lambrechtslab.sites.vib.be/en/single-cell. Additionally, the scRNA-seq data for seven treatment-naïve patients are available in the GEO (GSE176078). This manuscript does not report the original code. Any additional information required to reanalyze the data reported in this manuscript is available from the corresponding authors upon reasonable request.
