# Supplementary material for: Targeting SNRNP200-induced splicing dysregulation offers an immunotherapy opportunity for glycolytic triple-negative breast cancer
Source: Cell Discov. 2024 Sep 17;10:96. doi: 10.1038/s41421-024-00715-7 (PMC11405407; doi:10.1038/s41421-024-00715-7)
Supplement: Supplementary file 1 — Supplementary information [file 41421_2024_715_MOESM1_ESM.pdf]

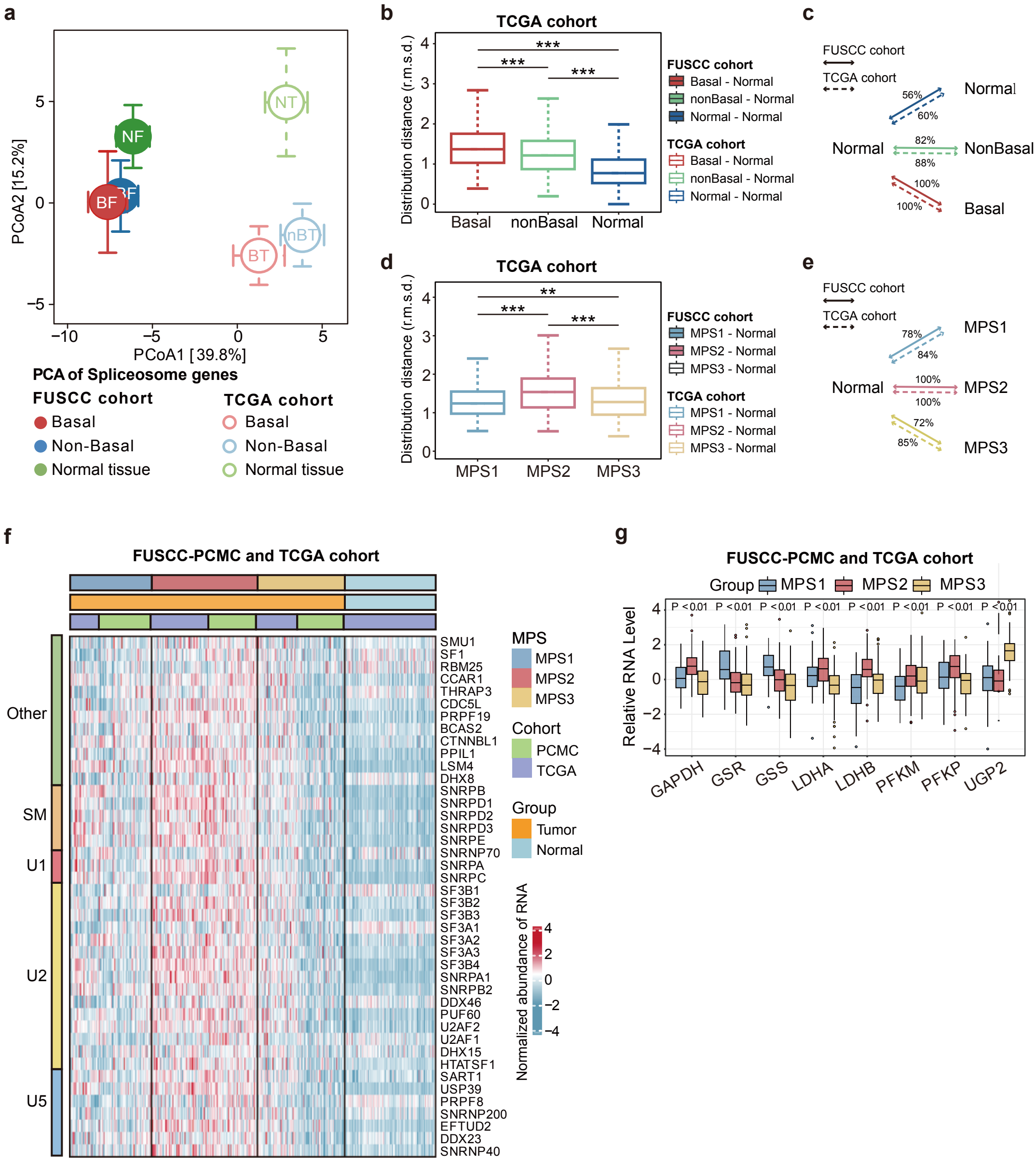

Supplementary Fig. S1. The landscape of RNA splicing is associated with metabolic dysregulation in TNBC.

a Principal coordinate analysis of spliceosome gene expression profiles in the FUSCC-TNBC (closed circles, n=360) and TCGA (open circles, n=261) cohorts. The circles and error bars indicate the means and standard errors of the means, respectively. **b** Global differences in spliceosome gene expression between basal, nonbasal, and normal tissues in the TCGA cohort. The distribution distances (r.m.s.d.) were calculated between basal tumors and corresponding normal tissues (red), nonbasal tumors and

corresponding normal tissues (green), and different samples of normal tissues (navy). P values were derived from the two-sided Wilcoxon rank-sum test and two-sided Kruskal–Wallis test (***, $p < 0.001$). The centerline indicates the median, and the bounds of the box indicate the 25th and 75th percentiles. **c** Inset summarizing average distances between tissue pairs as a percentage of the average distance between basal tumors and normal tissues in the FUSCC-TNBC (solid arrow) and TCGA cohorts (dotted arrow). **d** Global differences in spliceosome gene expression between MPS subtypes in the TCGA cohort. The distribution distances (r.m.s.d.) were calculated between MPS1 (blue), MPS2 (red), and MPS3 (yellow) tumors and their corresponding normal tissues. P values were obtained from the two-sided Wilcoxon rank-sum test and two-sided Kruskal–Wallis test (**, $p < 0.01$; ***, $p < 0.001$). The centerline indicates the median, and the bounds of the box indicate the 25th and 75th percentiles. **e** Inset summarizing average distances between tissue pairs as a percentage of the average distance between MPS2 tumors and normal tissues in the FUSCC-TNBC (solid arrow) and TCGA cohorts (dotted arrow). **f** Heatmap displaying normalized expression levels of 42 core spliceosome genes upregulated in tumors for each individual sample in the FUSCC-PCMC (n=179) and TCGA cohorts. **g** mRNA expression of GAPDH, GSR, GSS, LDHA, LDHB, PFKM, PFKP, and UGP2 in three MPS subtypes in the FUSCC-PCMC and TCGA cohorts (Wilcoxon test). The centerline indicates the median, and the bounds of the box indicate the 25th and 75th percentiles. BF, basal subtype in the FUSCC cohort; nBF, nonbasal subtype in the FUSCC cohort; NF, normal tissue in the FUSCC cohort; BT, basal subtype in the TCGA cohort; nBT, nonbasal subtype in the TCGA cohort; NT, normal tissue in the TCGA cohort.

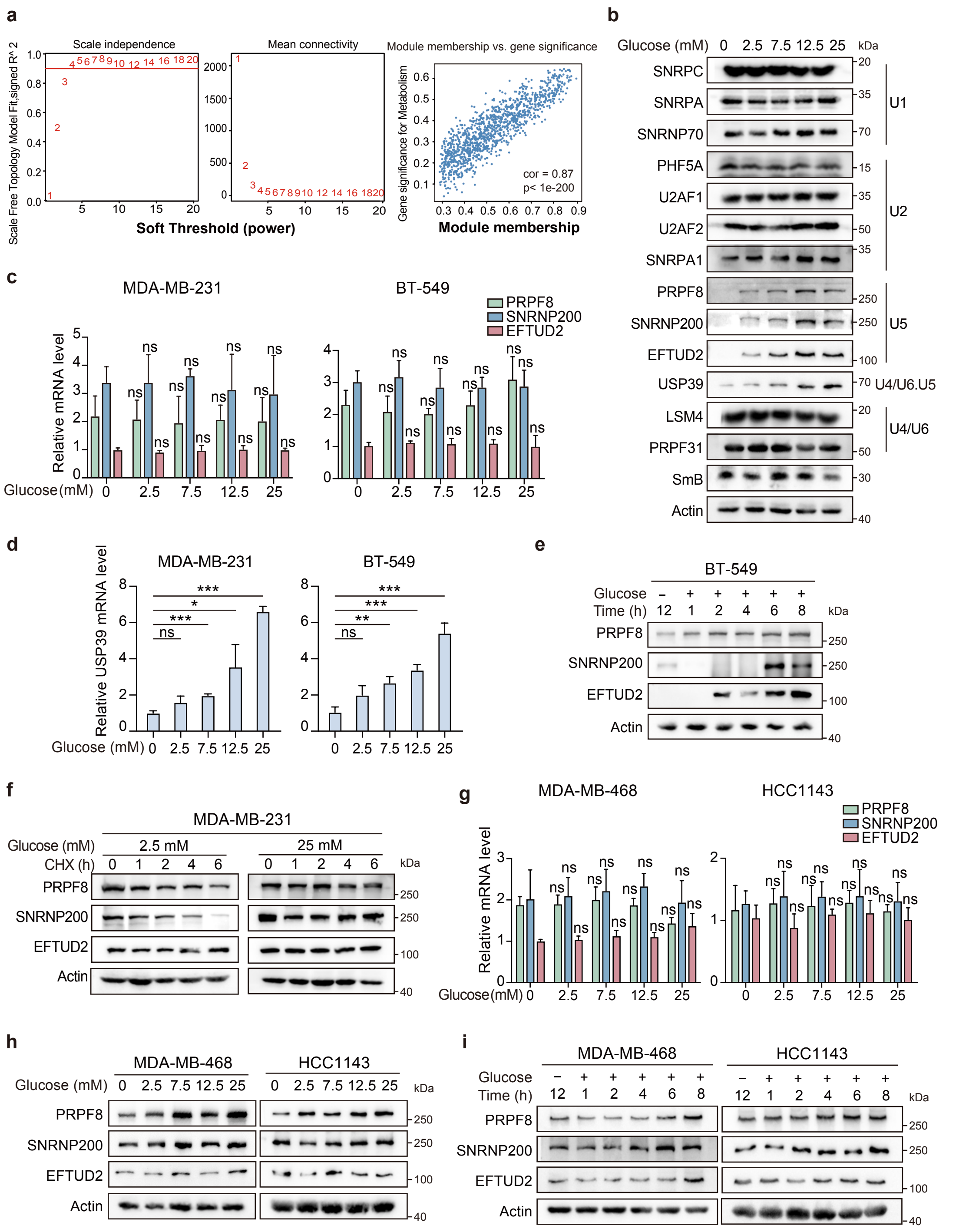

Supplementary Fig. S2. SNRNP200 is a crucial regulator in glycolytic TNBCs.

a Left: the scale-free fit index and mean connectivity (y-axis) as a function of soft-thresholding power (x-axis). Right: scatterplot depicting the relationship between gene significance and module membership for MPS2 module genes. Notably, genes within

the MPS2 module exhibited a substantial correlation with gene significance (correlation 0.87; $p < 0.0001$). **b** BT-549 cells were cultured in media supplemented with various concentrations of glucose for 16 hours, followed by immunoblotting analysis. **c** Quantitative PCR (qPCR) analysis of SNRNP200, EFTUD2, and PRPF8 mRNA levels in MDA-MB-231 cells (left) and BT-549 cells (right) cultured under different glucose concentrations for 16 hours. Comparisons of the mean mRNA levels of SNRNP200, EFTUD2, and PRPF8 under different glucose concentrations were made using Student's t test (ns, not significant, $p > 0.05$). **d** qPCR analysis of USP39 mRNA levels in MDA-MB-231 cells (left) and BT-549 cells (right) cultured under different glucose concentrations for 16 hours. The mean values were compared using Student's t test (**, $p < 0.01$; ***, $p < 0.001$). **e** BT-549 cells were glucose-starved for 12 hours and then stimulated with 25 mM glucose for various durations. The cell lysates were subjected to immunoblotting. **f** MDA-MB-231 cells maintained in 25 mM or 2.5 mM glucose were treated with cycloheximide (CHX, 10 mg/mL) for various durations, followed by immunoblotting analysis of the SNRNP200, EFTUD2, and PRPF8 protein levels. **g** qPCR analysis of SNRNP200, EFTUD2, and PRPF8 mRNA levels in MDA-MB-468 (left) and HCC1143 (right) cells cultured under different glucose concentrations for 16 hours. Comparisons of the mean mRNA levels of SNRNP200, EFTUD2, and PRPF8 under different glucose concentrations were made using Student's t test (ns, not significant, $p > 0.05$). **h** MDA-MB-468 (left) and HCC1143 (right) cells were cultured in media supplemented with various concentrations of glucose for 16 hours, followed by immunoblotting analysis. **i** MDA-MB-468 (left) and HCC1143 (right) cells were glucose starved for 12 hours and then stimulated with 25 mM glucose for various durations. The cell lysates were subjected to immunoblotting.

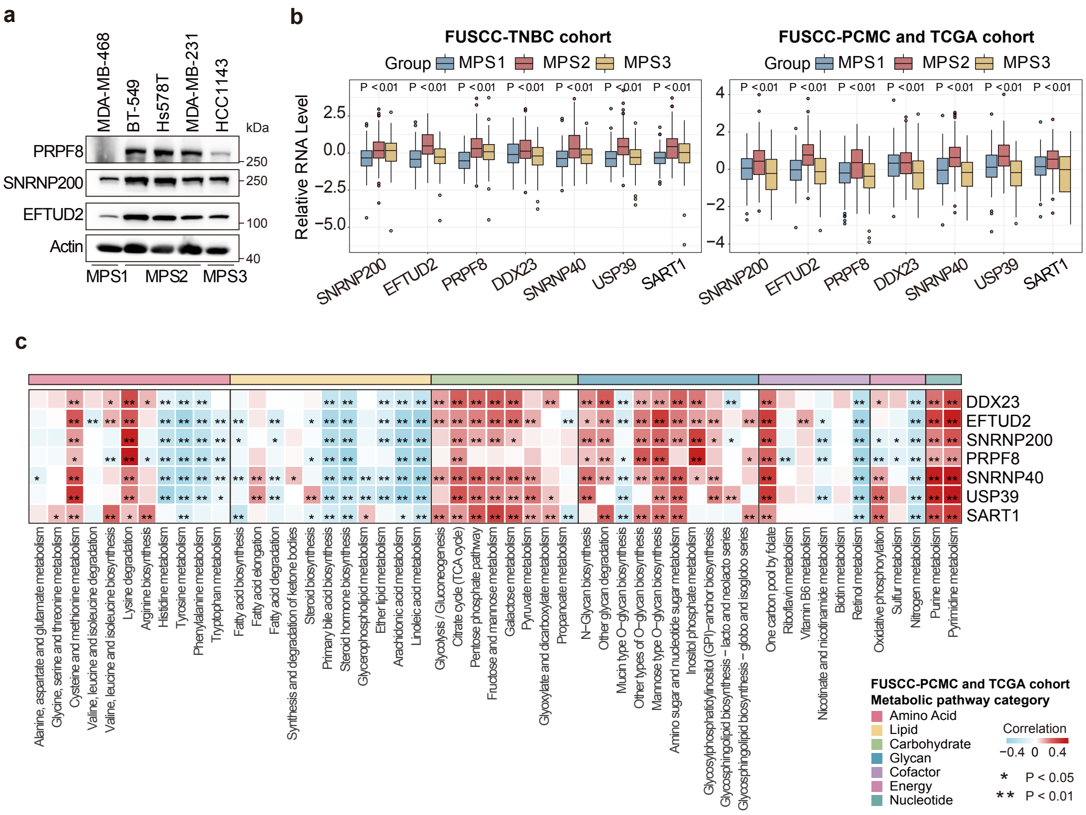

Supplementary Fig. S3 U5 snRNP components were selectively upregulated in MPS2 TNBCs.

a Representative Western blot analysis showing SNRNP200, EFTUD2, and PRPF8 protein levels in a panel of breast cancer cell lines. **b** mRNA expression levels of U5 snRNP and U4/U6. U5 tri-snRNP components, including SNRNP200, EFTUD2, PRPF8, DDX23, SNRNP40, USP39, and SART1, across three MPS subtypes in the FUSCC-TNBC cohort (left) and comparison with the FUSCC-PCMC and TCGA cohorts (right; Wilcoxon test). The centerline indicates the median, and the bounds of the box indicate the 25th and 75th percentiles. **c** Correlation coefficient heatmap illustrating associations between normalized mRNA expression levels of upregulated U5 snRNP genes in tumors and enrichment scores of metabolic pathways using Spearman's correlation analysis in the FUSCC-TNBC and FUSCC-PCMC cohorts (*, $p < 0.05$; **, $p < 0.01$).

a

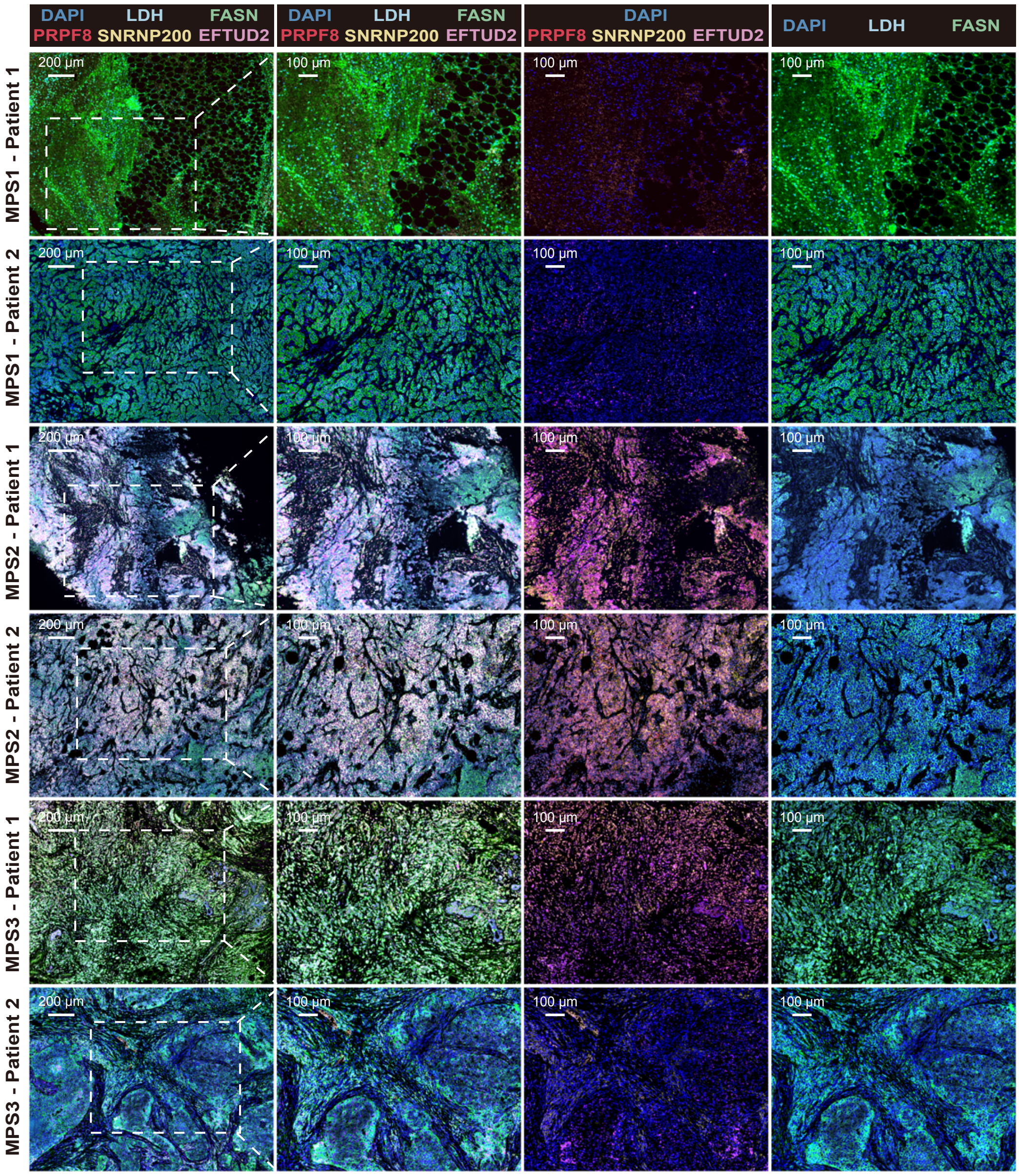

Supplementary Fig. S4 Multiplex Immunofluorescence for MPS Subtypes

a Representative images showing multiplex immunofluorescence staining for the indicated markers in each MPS of TNBC patients. Scale bars, 200 μm or 100 μm as indicated.

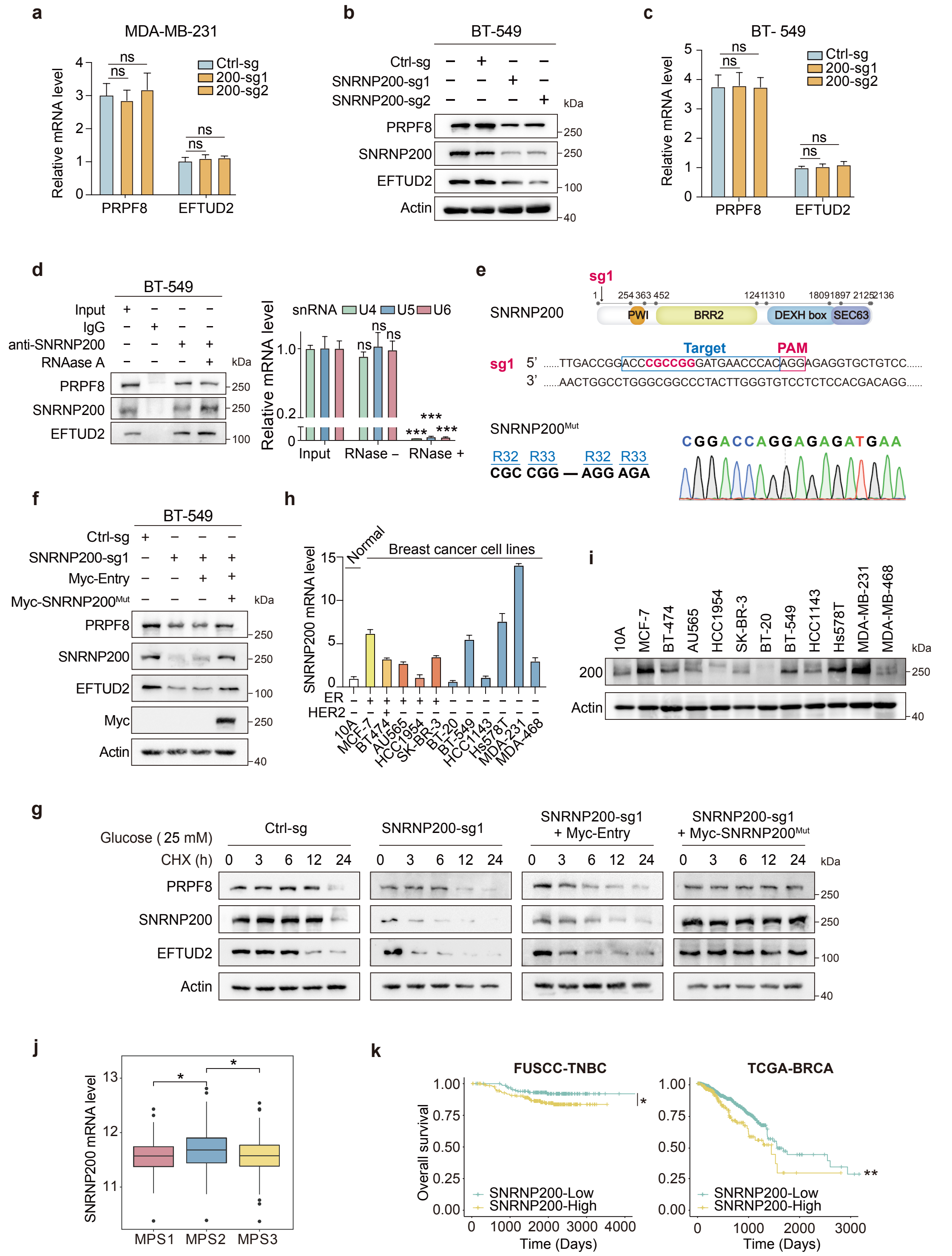

Supplementary Fig. S5 SNRNP200 is required for U5 snRNP complex stability.

a qPCR analysis of EFTUD2 and PRPF8 expression levels in control and SNRNP200-knockdown MDA-MB-231 cells. The mean values were compared using Student's t test (ns, not significant, p > 0.05). **b** Western blot analysis of SNRNP200, EFTUD2,

and PRPF8 expression levels in control and SNRNP200-knockdown BT-549 cells. **c** qPCR analysis of EFTUD2 and PRPF8 expression levels in control and SNRNP200-knockdown BT-549 cells. The mean values were compared using Student's t test (ns, not significant; $p > 0.05$). **d** BT-549 cells were immunoprecipitated with an anti-SNRNP200 antibody, with IgG serving as a negative control. The immunoprecipitation assays were analyzed by Western blot analysis (left). qPCR analysis of the U4, U5 and U6 snRNA levels in the input, RNase A-, and RNase A+ groups (right). The relative U4, U5, and U6 snRNA levels were normalized and compared to the input values via the 2^{-Ct} method (ns, not significant; $p > 0.05$; ***, $p < 0.001$). **e** The SNRNP200 synonymous substitution mutation affecting positions 32 and 33 ($SNRNP200^{Mut}$) prevented the knockdown by SNRNP200 sgRNA-1. Generation of plasmids harboring R32 and R33 mutations in the SNRNP200 gene. **f** SNRNP200-knockdown and control BT-549 cells were transiently transfected with the indicated plasmids, followed by immunoblotting analysis. **g** SNRNP200-knockdown and control cells were transiently transfected with the indicated plasmids and treated with cycloheximide (CHX, 10 mg/mL). The cells were harvested at 0, 3, 6, 12, and 24 hours, after which the protein levels were analyzed by Western blotting. **h, i** Quantitative PCR (qPCR) analysis of SNRNP200 mRNA levels (**h**) and representative Western blot analysis (**i**) in normal breast epithelial cells (10A) and a breast cancer cell panel. **j** Normalized SNRNP200 mRNA levels in three MPS subtypes in the FUSCC-TNBC cohort (Wilcoxon test, *, $p < 0.05$). The centerline indicates the median, and the bounds of the box indicate the 25th and 75th percentiles. **k** Kaplan–Meier curves of OS for patients in the FUSCC-TNBC cohort and TCGA-BRCA cohort (n=1096) according to the log-rank test (*, $p < 0.05$; **, $p < 0.01$).

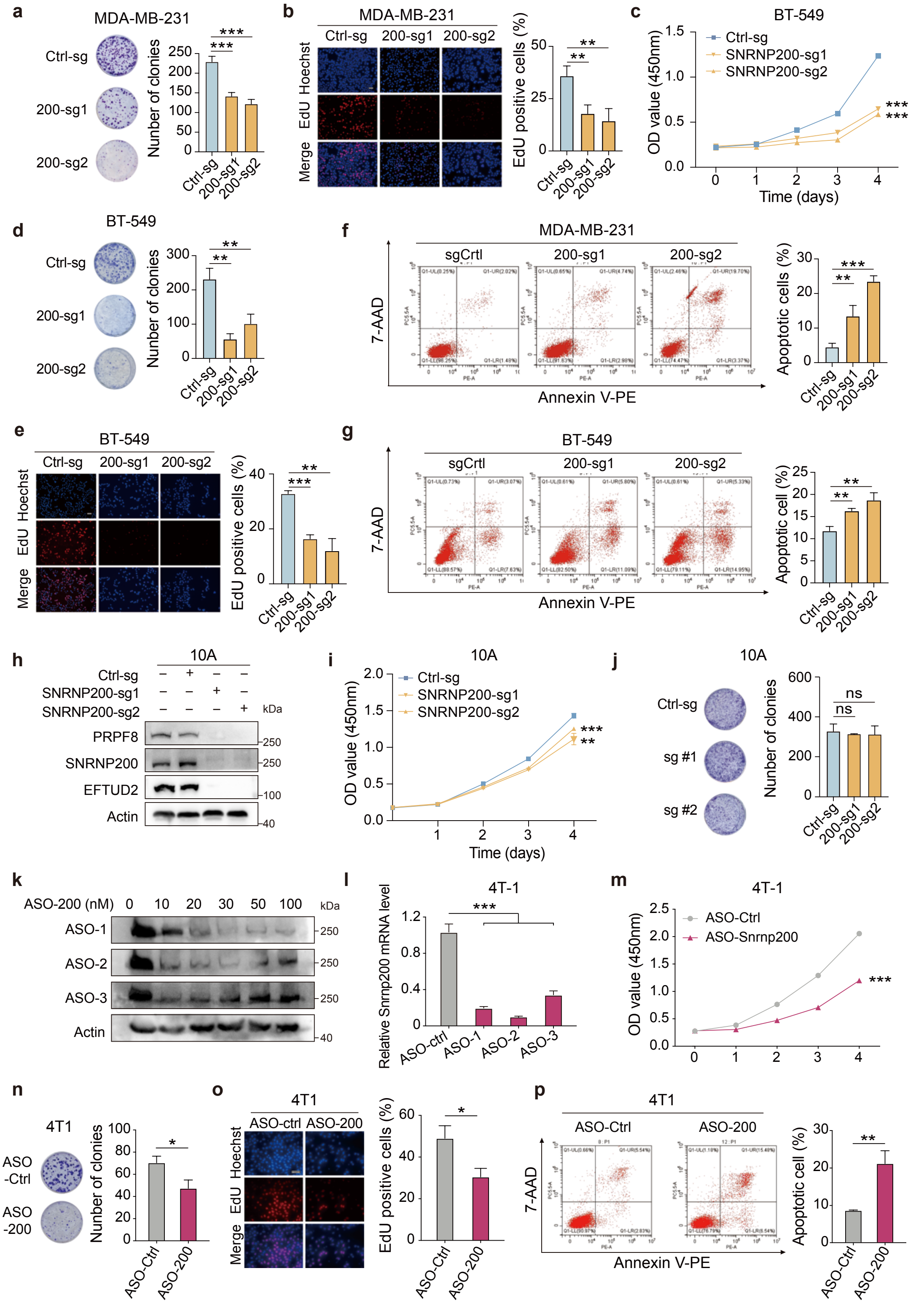

Supplementary Fig. S6 SNRNP200 promotes tumor proliferation *in vitro* and *in vivo*.

a, b Colony formation (**a**) and EdU incorporation (**b**) assays in control and SNRNP200-

knockdown MDA-MB-231 cells. Scale bars, 50 μm. **c**-**e** CCK-8 (**c**), colony formation (**d**), and EdU incorporation (**e**) assays in control and SNRNP200-knockdown BT-549 cells. Scale bars, 50 μm. **f, g** Apoptosis of control and SNRNP200-knockdown MDA-MB-231 (**f**) and BT-549 cells (**g**) was evaluated via Annexin V-PE/7-AAD staining. **h** Western blot analysis of the SNRNP200, EFTUD2 and PRPF8 levels in control and SNRNP200-knockdown MCF10A cells. **i, j** CCK-8 proliferation (**i**) and colony formation (**j**) assays in control and SNRNP200-knockdown MCF10A cells. **k** Immunoblotting analysis of 4T1 cells transfected with ASOs at various concentrations (0 nM, 10 nM, 20 nM, 30 nM, 50 nM, and 100 nM) with mouse Snrnp200 antibodies. **l** qPCR analysis of Snrnp200 expression levels in 4T1 cells transfected with ASOs targeting Snrnp200 or the control (30 nM). **m**-**o** CCK-8 (**m**), colony formation (**n**), and EdU incorporation (**o**) assays of 4T1 cells transfected with ASOs targeting Snrnp200 or the control. **p** The apoptosis of 4T1 cells transfected with ASOs targeting Snrnp200 or the control ASO was evaluated by Annexin V-PE/7-AAD staining. The difference between two groups was calculated using two-tailed Student's t test (ns, not significant, $p > 0.05$; *, $p < 0.05$; **, $p < 0.01$; ***, $p < 0.001$).

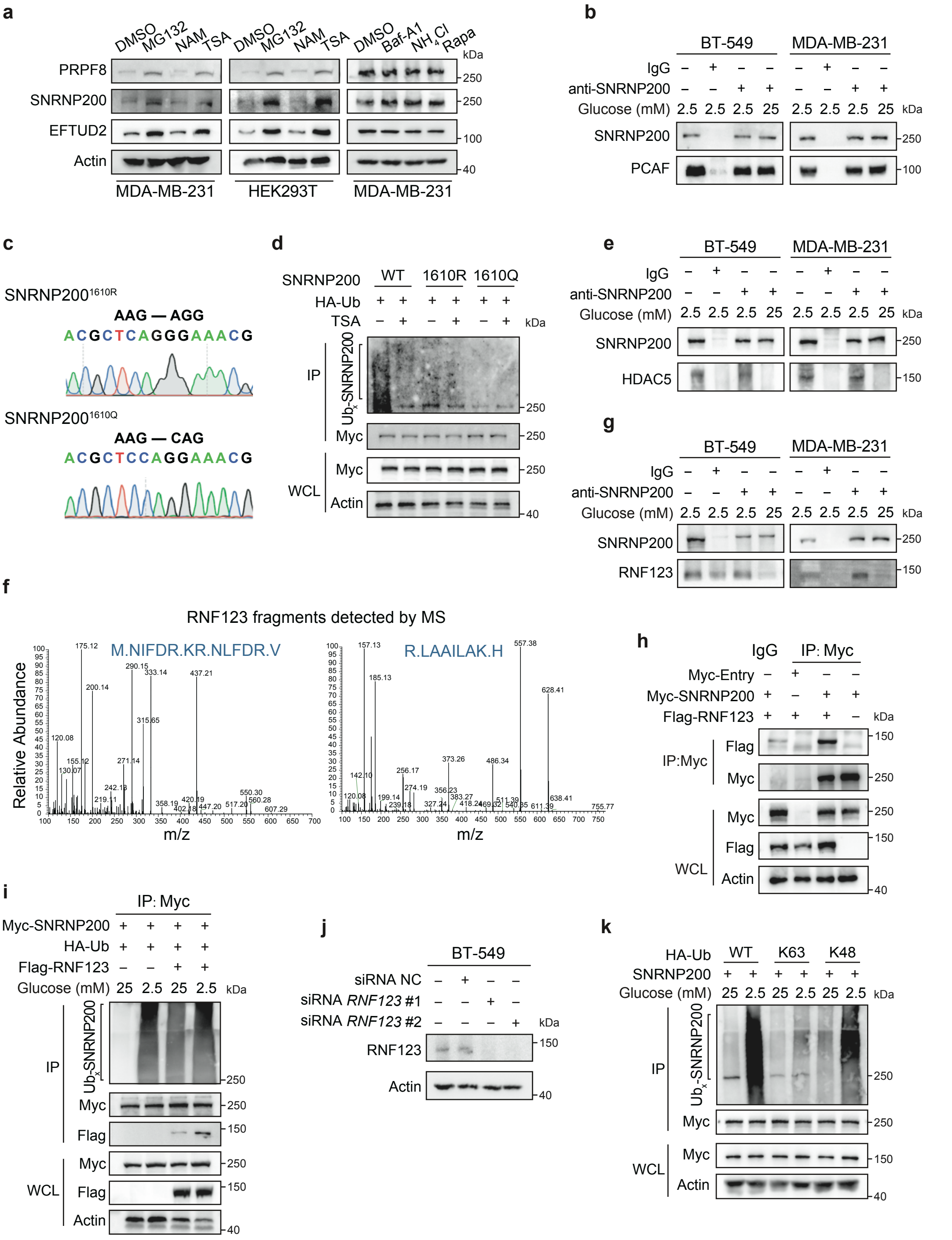

Supplementary Fig. S7. PCAF interacts with SNRNP200 and acetylates SNRNP200 at K1610.

a Immunoblot analysis of SNRNP200, EFTUD2, and PRPF8 protein levels in MDA-MB-231 and HEK293T cells treated with or without nicotinamide (NAM, 5 mM, 6

hours), trichostatin A (TSA, 10 μM, 16 hours), Baf-A1 (200 nM, 16 hours), NH4Cl (20 mM, 16 hours) or rapamycin (Rapa, 1 μM, 16 hours). MG132 treatment (10 μM, 12 hours) was used as a positive control. **b** Immunoprecipitation assays of SNRNP200 in BT-549 and MDA-MB-231 cells using an anti-SNRNP200 antibody, followed by Western blot analysis with SNRNP200 and PCAF antibodies. **c** Generation of plasmids harboring K1610 mutations in the SNRNP200 gene. **d** HEK293T cells transfected with the indicated plasmids with or without TSA treatment were subjected to ubiquitylation analysis, as revealed by immunoblotting. **e** Immunoprecipitation assays of SNRNP200 in BT-549 and MDA-MB-231 cells, followed by Western blot analysis with SNRNP200 and HDAC5 antibodies. **f** LC–MS analysis of endogenous RNF123 immunoprecipitated with an anti-SNRNP200 antibody from HEK293T cells, showing MS2 spectra for two signature peptides of RNF123. **g** Immunoprecipitation assays of SNRNP200 in MDA-MB-231 and BT-549 cells, followed by Western blot analysis with SNRNP200 and RNF123 antibodies. **h** Coimmunoprecipitation assays of Myc-tagged SNRNP200 and Flag-tagged RNF123 in HEK293T cells using an anti-Myc-tag antibody. **i** Immunoblot analysis of SNRNP200 ubiquitylation levels in HEK293T cells treated with varying glucose concentrations with coimmunoprecipitation assays to assess the dynamic interactions between RNF123 and SNRNP200. **j** Western blot analysis of RNF123 expression levels in control and RNF123-depleted BT-549 cells. **k** Immunoblot analysis of the effect of glucose concentration on the SNRNP200 ubiquitin chain type in HEK293T cells transfected with the indicated constructs.

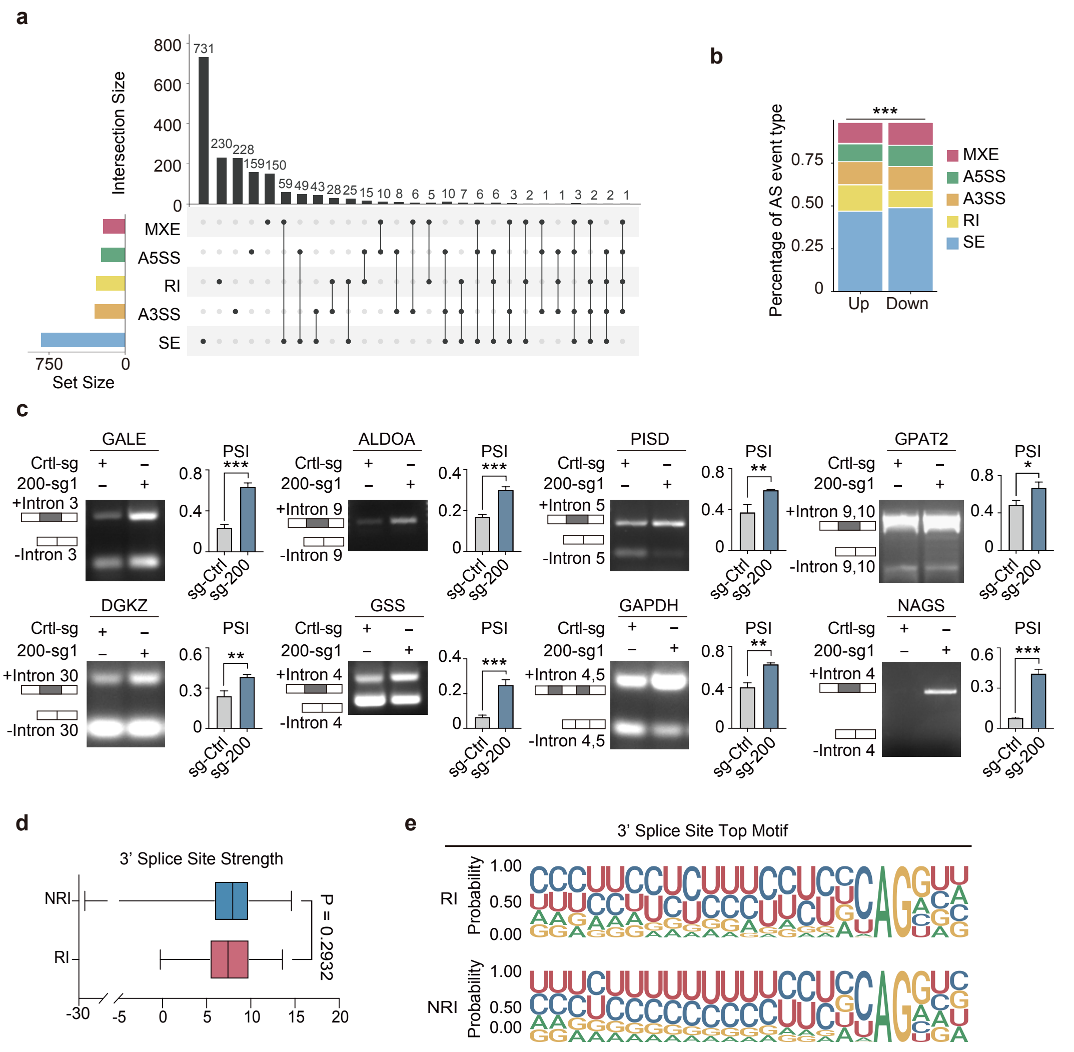

Supplementary Fig. S8. SNRNP200 enhances the RNA splicing of metabolic enzyme-encoding genes.

a UpSet graphic of five types of alternative splicing (AS) events in SNRNP200-knockdown MDA-MB-231 cells. **b** Percentage distribution of five types of AS events in SNRNP200-knockdown MDA-MB-231 cells. Statistical significance was determined using the chi-square test (***, p < 0.001). **c** Representative RT–PCR validation of SNRNP200-regulated AS events in BT-549 cells. Diagrams illustrating the structure of each isoform. Individual data points are shown (n = 3; *, p < 0.05; **, p < 0.01; ***, p < 0.001; Student's t test). **d** Analysis of 3′ splice site strength across retained introns (RIs) compared with nonretained introns (NRIs) within the same set of genes (Wilcoxon rank-sum test). **e** Motif enrichment analysis of the same set of genes,

highlighting the most frequently identified motifs aligned with the consensus 3′ splice site sequences for both the RI and NRI.

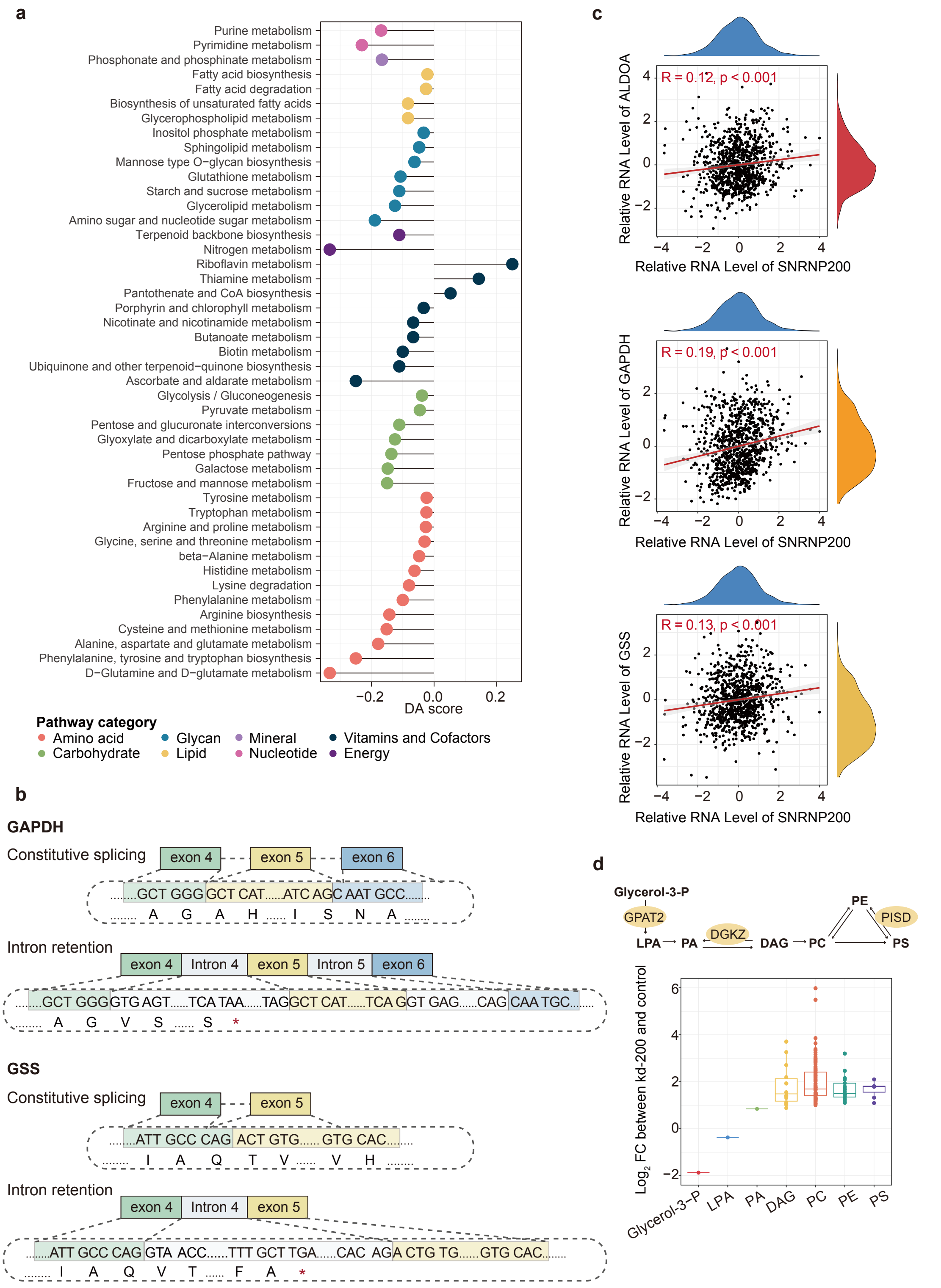

Supplementary Fig. S9 SNRNP200 regulates cell metabolism via RNA splicing.

a The differential abundance (DA) score was used to quantify alterations in metabolic pathways by comparing metabolite abundances between control and SNRNP200-knockdown MDA-MB-231 cells. This score indicates the overall change in metabolite

levels within a pathway. A DA score of 1 signifies an increase in all measured metabolites in the pathway following SNRNP200 knockdown, whereas a score of -1 indicates a decrease. Pathways with at least three measured metabolites were considered for calculating the DA score. **b** The top schematic diagram illustrates two GAPDH transcripts (normal splicing and intron retention), whereas the bottom schematic diagrams depict GSS transcripts (normal splicing and intron retention). The detailed mRNA and encoded protein sequences are shown to indicate the specific site in which the protein is truncated. **c** Scatterplot showing the correlation between the normalized RNA levels of SNRNP200 and metabolic enzyme-encoding genes, including ALDOA, GAPDH, and GSS, in TNBC patients from the FUSCC-TNBC, FUSCC-PCMC, and TCGA cohorts. The red line represents the fitting curve calculated by Pearson correlation. **d** The diagram provides a visual representation of SNRNP200-regulated lipids, accompanied by their corresponding upstream metabolic enzymes (depicted as circles) that undergo intron retention following SNRNP200 ablation (top). Log_2-fold changes in the abundances of different lipids in control and SNRNP200-knockdown MDA-MB-231 cells (bottom). FA, fatty acid; GL, glycerolipid; GP, glycerophospholipid; SP, sphingolipid; ST, sterol lipid; Glycerol-3-P, glycerol 3-phosphate; LPA, lysophosphatidic acid; PA, phosphatidic acid; DAG, diacylglycerol; PC, phosphatidylcholine; PE, phosphatidylethanolamine; PS, phosphatidylserine.

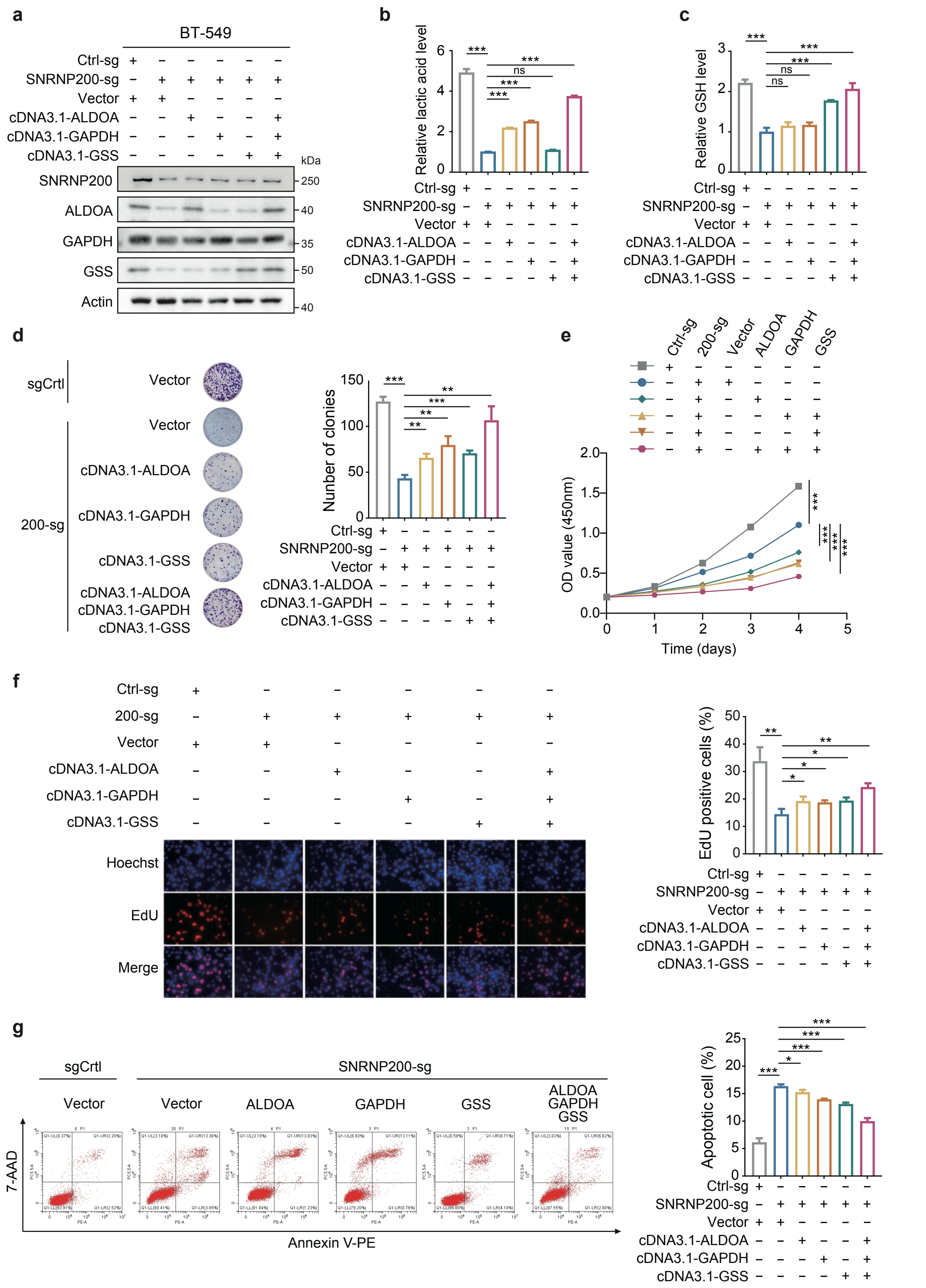

Supplementary Fig. S10. SNRNP200 promotes MPS2 TNBC progression through its regulation of cellular metabolism.

a Rescue assay showing the introduction of exogenous ALDOA, GAPDH, and GSS into SNRNP200-depleted BT-549 cells. The expression levels of ALDOA, GAPDH,

and GSS were analyzed by Western blot analysis. **b, c** Relative lactic acid (**b**) and glutathione (GSH) levels (**c**) in SNRNP200-depleted BT-549 cells transiently transfected with the indicated plasmids. The data are presented as the means ± SEMs. **d-f** Colony formation assay (**d**), CCK-8 assay (**e**), and EdU incorporation assay (**f**) in control or SNRNP200-depleted BT-549 cells transiently transfected with the indicated plasmids. Scale bars, 50 μm. **g** Apoptosis of SNRNP200-depleted BT-549 cells transiently transfected with the indicated plasmids, as assessed by Annexin V-PE/7-AAD staining. The difference between two groups was calculated using two-tailed Student's t test (ns, not significant, $p > 0.05$; *, $p < 0.05$; **, $p < 0.01$; ***, $p < 0.001$).

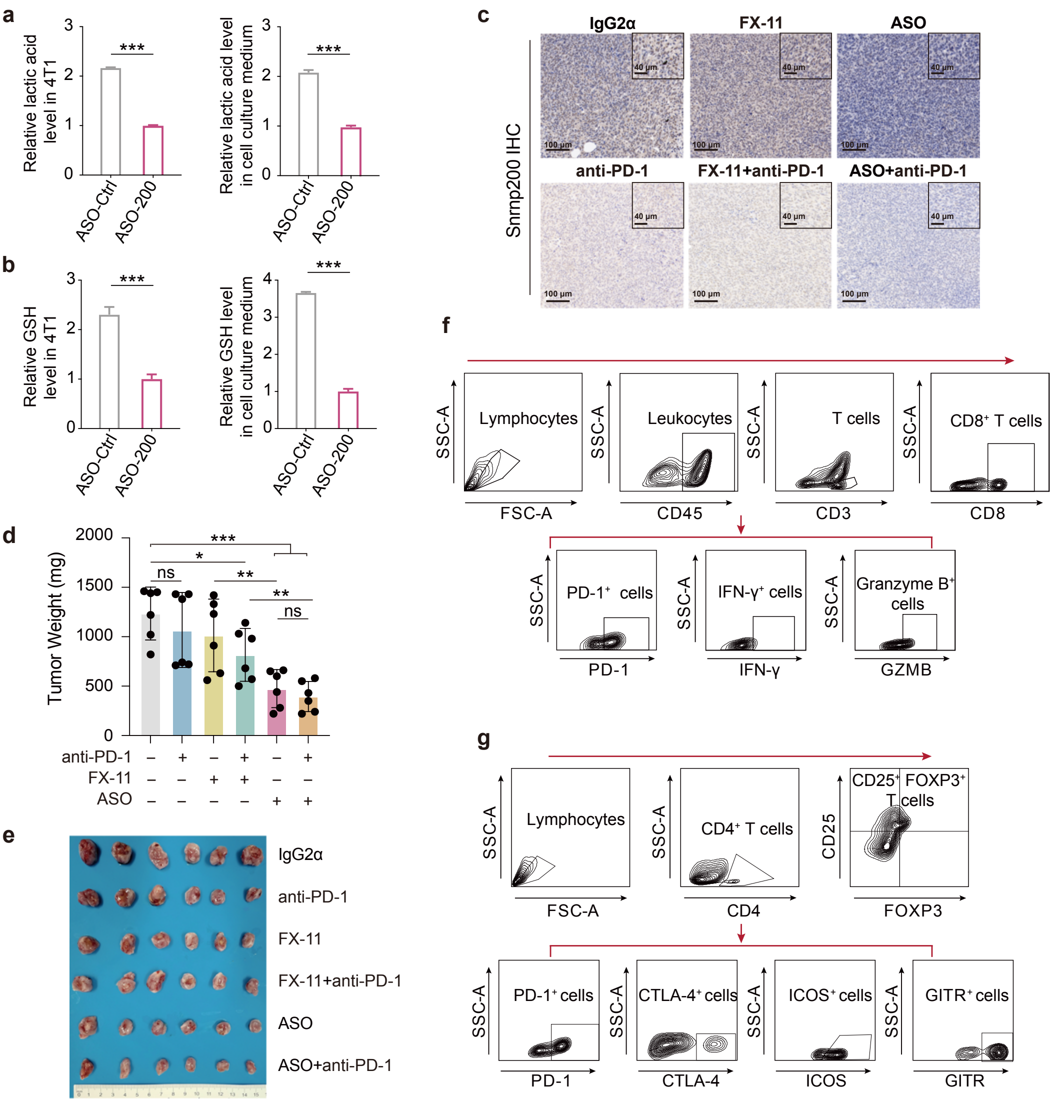

Supplementary Fig. S11. ASO-Snrnp200 plus immunotherapy inhibited glycolytic tumor growth.

a, b Relative lactic acid (**a**) and GSH (**b**) levels in 4T1 cells and cell culture media after transfection with ASOs targeting Snrnp200 or the control. The data are presented as the means ± SEMs. Statistical analysis was performed using Student's t test (***, $p < 0.001$). **c** Representative immunochemical images of Snrnp200 in the six groups. Scale bars, 40 μm. **d** Tumor weights of the six treatment groups. The data are presented as the means ± SEMs. The difference between two groups was calculated using two-tailed Student's t test (ns, not significant, $p > 0.05$; *, $p < 0.05$; **, $p < 0.01$; ***, $p < 0.001$). **e** Representative image of tumors illustrating the effect of Snrnp200 ASO plus PD-1 blockade (n=6 mice/group). **f** The gating strategy for flow cytometry analysis of

lymphocytes included leucocytes ($CD45^+$), T cells ($CD45^+CD3e^+$), $CD8^+$ T cells ($CD45^+CD3e^+CD8^+$), PD-1^+ $CD8^+$ T cells ($CD45^+CD3e^+CD8^+$PD-1^+), IFN-$\gamma^+$$CD8^+$ T cells ($CD45^+CD3e^+CD8^+$IFN-γ^+) and granzyme B^+ $CD8^+$ T cells ($CD45^+CD3e^+CD8^+GZMB^+$). **g** Gating strategy for flow cytometry analysis of lymphocytes, $CD4^+$ T cells ($CD4^+$), Treg cells ($CD4^+CD25^+FOXP3^+$), PD-1^+ Treg cells ($CD4^+CD25^+FOXP3^+$PD-1^+), CTLA-4+ Treg cells ($CD4^+CD25^+FOXP3^+$CTLA-4^+), $ICOS^+$ Treg cells ($CD4^+CD25^+FOXP3^+ICOS^+$) and $GITR^+$ Treg cells ($CD4^+CD25^+FOXP3^+$ $GITR^+$). For figures **f** and **g**, the numbers in the graphs indicate the percentage of cells. Plots of data from one representative tumor are shown.

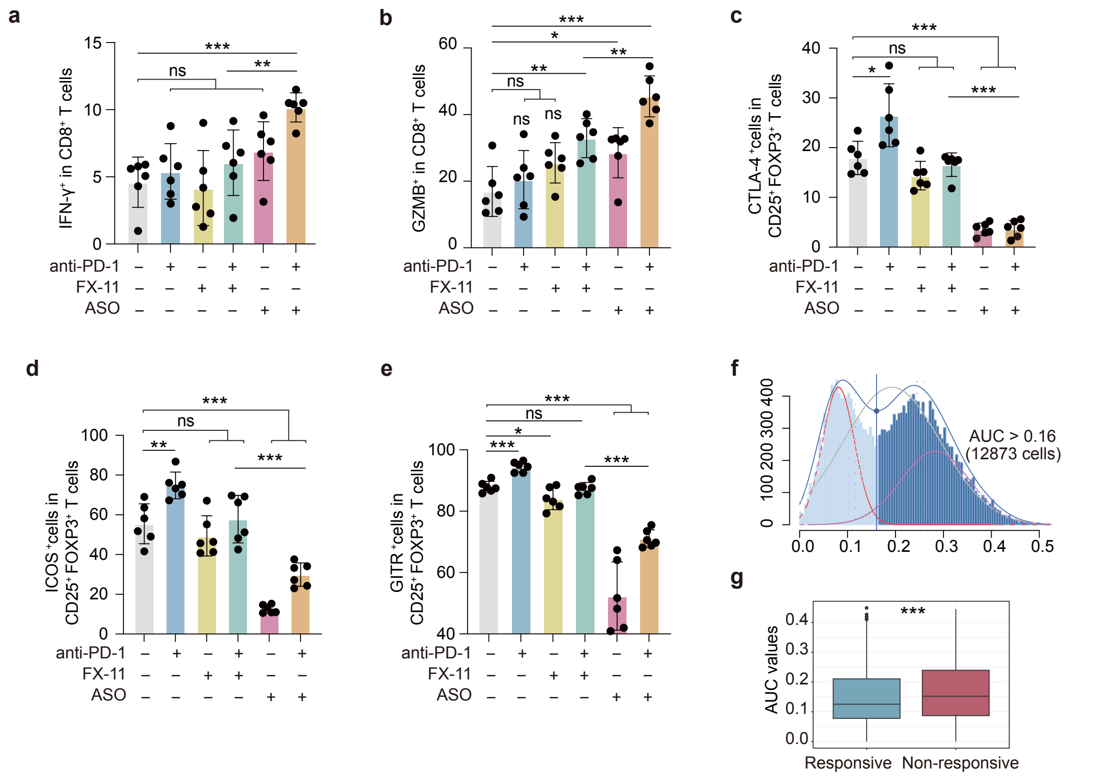

Supplementary Fig. S12. ASO-Snrnp200 enhances the efficiency of immunotherapy inhibition in glycolytic MPS2 tumors.

a, b Bar plots showing the percentages of IFN-γ^+ (**a**) and GZMB$^+$ cells (**b**) among CD8$^+$ T cells. **c-e** Bar plots showing the percentages of CTLA4$^+$ (**c**), ICOS$^+$ (**d**) and GITR$^+$ (**e**) cells among Treg cells. **f** AUC values for gene sets comprising 42 upregulated spliceosome genes in glycolytic TNBC. A threshold of 0.16 was applied, and the AUC of 12873 cancer cells surpassed this threshold. **g** Comparison of AUC values in cancer cells between patients who responded and those who did not respond to immunotherapy. The data are presented as the means ± SEMs and were compared using Student's t test; ***, $p < 0.001$.
